# Long‐Tea‐CLIP: An Expert‐Level Multimodal AI Framework for Fine‐Grained Green Tea Grading Across Five Sensory Dimensions

**DOI:** 10.1002/advs.202518235

**Published:** 2026-03-27

**Authors:** Yanqun Xu, Zhengfang Xue, Qing Luo, Xu Liu, Yongquan Xu, Jing Huang, Xingcai Zhang, Xinyuan Zhang, Zhonghua Liu, Zisheng Luo

**Affiliations:** ^1^ Department of Food Science & Technology School of Agriculture and Biology Shanghai Jiao Tong University Shanghai China; ^2^ National Research Center of Engineering and Technology for Utilization of Botanical Functional Ingredients Hunan Agricultural University Changsha China; ^3^ College of Biosystems Engineering and Food Science Zhejiang University Hangzhou China; ^4^ Laboratory of Agro‐Products Postharvest Handling of Ministry of Agriculture and Rural Affairs Zhejiang Key Laboratory of Agri‐Food Resources and High‐Value Utilization Innovation Center for Postharvest Agro‐Products Technology Zhejiang University Hangzhou China; ^5^ The Rural Development Academy Zhejiang University Hangzhou China; ^6^ School of Intelligent Systems Science and Engineering Jinan University Zhuhai China; ^7^ China Tea Science Society Hangzhou China; ^8^ Tea Research Institute Chinese Academy of Agricultural Sciences Key Laboratory of Tea Biology and Resources Utilization Ministry of Agriculture Hangzhou China; ^9^ Department of Materials Science and Engineering Stanford University Stanford California USA

**Keywords:** computer vision, Contrastive Language‐Image Pre‐training, green tea grading, metabolomics, multimodal AI, sensory evaluation

## Abstract

Traditional tea quality evaluation depends on human evaluators, limiting scalability, and consistency. To establish an artificial intelligence (AI)‐assisted framework for comprehensive evaluation of tea quality and detailed assessment of the tea consumption experience, this study aims to develop Long‐Tea‐CLIP (Contrastive Language‐Image Pre‐training), a multimodal tea grading system that combines computer vision and chemoinformatics. It integrates five sensory evaluation dimensions for green tea, using separate submodels for appearance (ResNet‐18), soup color (eXtreme Gradient Boosting (XGBoost)), aroma, infused leaf, and taste (multilayer perceptron (MLP)). A deep network derived from ResNet‐18 integrates dry tea images with seven subdimensions of sensory comments to achieve a refined appearance “grading.” We apply Tip‐CLIP supervised MLP on feature data extraction from infused leaf and chemical data of aroma and taste to enhance accuracy. Submodel outputs are weighted into a unified framework to produce an overall score. Long‐Tea‐CLIP trained on 7763 image‐text pairs from 38 Longjing tea varieties achieves 92% accuracy, indicating its potential to enhance tea quality control and market transparency.

## Graphical abstract

Long‐Tea‐CLIP (Contrastive Language‐Image Pre‐training) presents a multimodal AI framework that integrates visual, metabolomic, and sensory knowledge to grade green tea across appearance, soup color, aroma, taste, and infused leaf. By combining expert‐guided modeling with CLIP‐supervised learning, the system delivers fine‐grained quality evaluation and improves the consistency, interpretability, and scalability of tea grading.

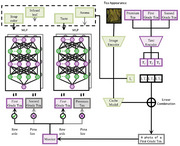

## 1 Introduction

The grading of agricultural products enhances overall market competitiveness and increases sales prices. However, traditional manual grading methods are constrained by high labor intensity, elevated costs, low efficiency, and subjectivity. To overcome these challenges, automated grading technologies, particularly those based on machine learning and computer vision, have been extensively investigated. Tea, the most consumed beverage globally [1], is renowned for its flavor and health benefits [2, 3]. The International Standard ISO 20715 classifies tea into black, green, yellow, white, oolong, and dark, with green tea representing the largest production, accounting for >50% [4]. FAO's (Food and Agriculture Organization of the United Nations) statistics of global green tea production for the year 2022 is as high as 6995979.72 *t*, making it a crucial economic sector [5].

Compared to other agricultural products, tea grading is more complex as it requires evaluation across multiple dimensions. The criteria for tea evaluation vary significantly among different countries, yet typically revolve weighted calculations around the tea appearance and flavor, soup color, and infused leaf. In Japan, compared to external appearance, the internal sensory attributes such as aroma and taste are given higher priority, accounting for approximately 65%–70% of the total evaluation score, whereas appearance occupies only 10%–20%. Furthermore, Japanese tea evaluation usually does not involve assessment of the infused leaf. Similarly, the Korean Industrial Standard KS H 2161:2023 (green tea) does not emphasize the assessment of infused leaves. In China, aroma and taste together account for approximately 50% of the total scoring, and evaluation of the infused leaf is routinely considered for almost all tea categories. Recently, the international standard ISO 18449:2025, titled “Green tea—Vocabulary,” jointly formulated by tea standardization experts from China, the UK, Germany, Japan, Sri Lanka, Kenya, and other countries, also elaborates on the sensory characteristics of green tea by focusing specifically on the appearance of the dry tea leaf (including shape, color, cleanliness, and evenness), the appearance and odor of the infused leaf, and the color and taste of the tea liquor. For the sake of conciseness, we will refer to these five dimensions as appearance and infused leaf, aroma, soup color, and taste.

Traditionally, tea grading has predominantly depended on artificial sensory evaluation, a time‐consuming and labor‐intensive process prone to subjectivity and inconsistencies [6]. In extensive global market, whole‐leaf teas are frequently marketed based on their grades, making accurate and consistent grading essential. Tea is categorized into different grades based on appearance, aroma, taste, soup color, and infused leaf characteristics [7]. These grades influence consumers’ buying choices and significantly impact the economic value of the tea, highlighting the importance of precise quality assessment [8, 9]. It is difficult for ordinary consumers to separate the good and bad of commercial tea through the appearance or smell of tea leaves and other characteristics. Expert tea evaluators play a vital role in maintaining quality standards; the shortage of skilled evaluators and the inherent limitations of sensory analysis highlight the need for more objective, efficient, and reliable grading methods. Recent advancements in artificial intelligence (AI) offer promising solutions for data classification, a technique employed in diverse fields, intelligent diagnostics [10, 11], and personalized agriculture [12, 13]. Artificial Intelligence can replace human tea evaluators in the evaluation process with high consistency and reduced operator‐training costs. Studies have investigated machine learning algorithms and computer vision systems to examine specific tea characteristics [14, 15]. In addition to the appearance of tea leaves, studies have also begun to focus on how to differentiate the overall quality of the tea leaves including taste, aroma, and several other dimensions [16]. However, this type of method still omits some judging angles compared to human judges, such as soup color and infused leaf. This greatly affects the reliability of this method.

**FIGURE 1:**
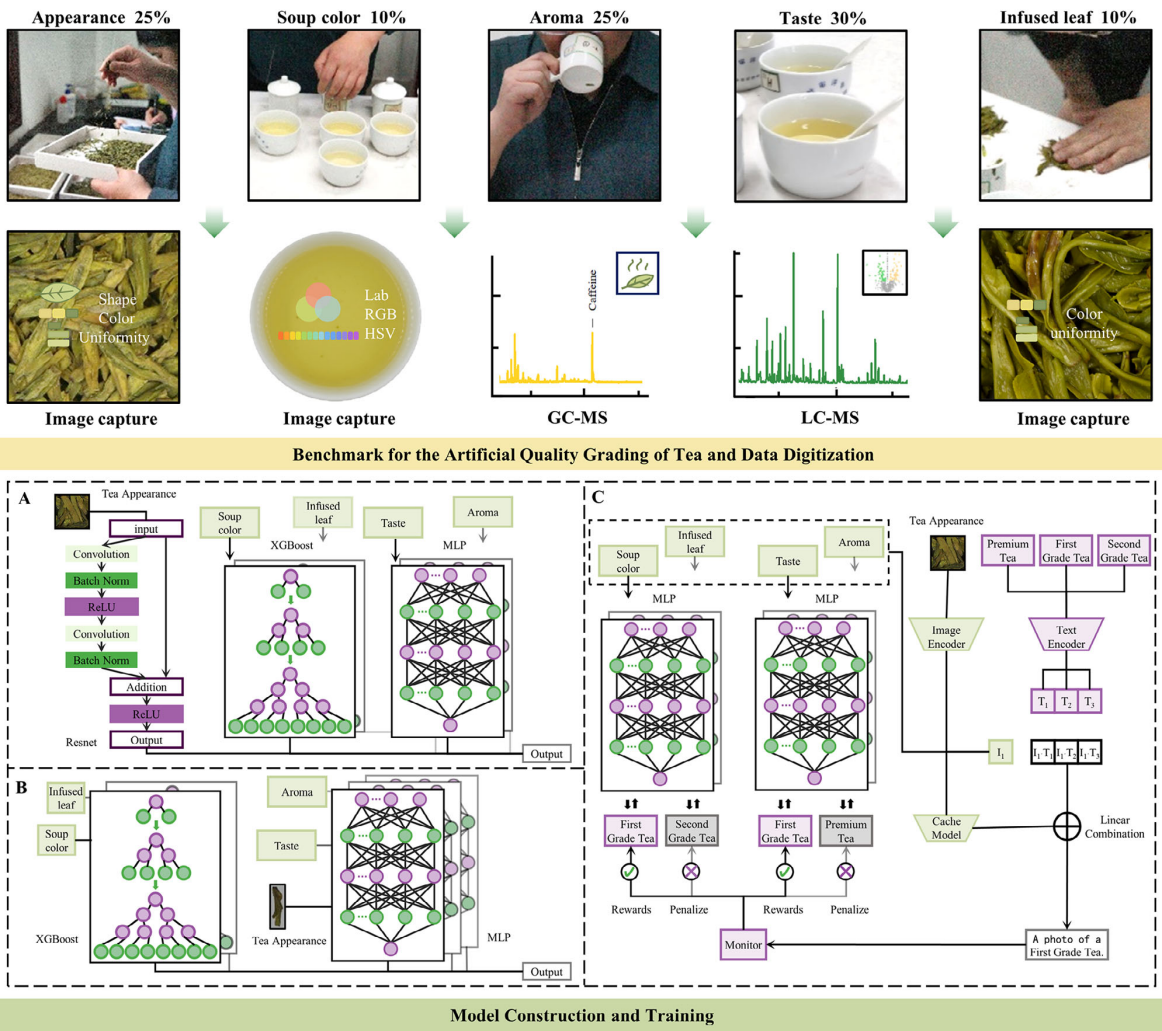
Overall workflow for digitizing and modeling the expert manual sensory evaluation of tea. The traditional evaluation dimensions—appearance, soup color, aroma, taste, and infused leaf—are, respectively, digitized and quantified using digital imaging of stacked dry tea, colorimetric imaging of tea broth, GC‐MS profiling, liquid chromatography–mass spectrometry (LC‐MS) profiling, and digital imaging of wet leaves. The acquired raw data undergo feature extraction and are subsequently fed into corresponding AI models. (a) Architecture of the Long‐Tea‐Intelligent module: ResNet is utilized to evaluate the appearance of stacked tea; XGBoost performs dimensional scoring for soup color and infused leaf; and multilayer perceptrons (MLPs) process taste and aroma data. The scores from these five dimensions are weighted and aggregated to derive the final grading score. (b) Architecture of the Long‐Tea‐Feature module: MLPs are employed for regression scoring based on explicitly extracted features from dispersed tea images, as well as taste and aroma profiles. XGBoost scores the soup color and infused leaf dimensions, followed by weighted aggregation. (c) Schematic of the Tip‐Clip Supervised Multi‐Layer Perceptron (TCSMLP) architecture, illustrating the supervised training of the MLP models using the multimodal CLIP framework as a supervisor to align sensory features with expert knowledge.

This study proposes a novel multimodal system for grading green tea. Our system, Long‐Tea (https://github.com/2521614022/Long‐Tea), utilizes AI to integrate visual, chemical, and sensory data, emulating the comprehensive assessment performed by expert tea evaluators. We utilize computer vision to acquire visual information on dry tea, soup color, and infused leaf; gas chromatography–mass spectrometry (GC‐MS) to analyze aroma profiles; and metabolomics data to analyze taste attributes. These data are integrated into a multimodal AI model Long‐Tea (based on Resnet [17, 18], eXtreme Gradient Boosting (XGBoost) [19], multilayer perceptron (MLP) [20], and Contrastive Language‐Image Pre‐training (CLIP)) [21]) trained on a dataset graded by internationally renowned tea masters. By combining these diverse data streams, we aim to develop a robust and accurate AI‐driven grading system that enhances quality control (QC), ensures consistency, and promotes transparency within the tea industry. Figure 1 depicts a graphical workflow of Long‐Tea.

## 2 Results

### 2.1 Benchmarking Multidimensional Quality Metrics as the Foundation for Tea Grading

International expert‐based sensory evaluation has long been the most recognized and authoritative system in tea grading. In order to obtain the overall and five subdimension scores and grades of tea samples, we invited five master tea evaluators to perform a standardized sensory assessment of 38 Longjing green tea samples. Comments and scores for each criterion were recorded, and a weighted sum was calculated to determine the total score (Table S2). In appearance, most samples exhibited the typical flat, straight appearance characteristics of Longjing tea; In aroma, six samples exhibited excessive roasting, negatively impacting their fresh aroma, and lowering aroma scores. Market preferences, processing methods, and ideologies of manufacturers influence roasting levels. Although high roasting was common in the past few years, most samples exhibited moderate roasting. In taste, several samples exhibited floral notes besides fresh aromas, lifting aroma scores. Ten samples exhibited floral notes, indicating a relatively high proportion. Longjing tea is assessed based on appearance, soup color, aroma, taste, and infused leaf, classified as appearance and intrinsic quality. Assuming proper processing appearance and intrinsic quality should be consistent. However, variations can cause discrepancies, where some teas with excellent appearance only achieve lower intrinsic quality grades. All samples’ scores ranged from 85 to 92 (Table S2), classified into three levels: premium grade (class A), first grade (class B), and second grade (class C). Total scores of <88 were classified as second grade, scores of ≥88 and <90 as first grade, and scores of ≥90 as premium grade. Among 38 samples, 16 were classified as class A, 16 as class B, and 6 as class C.

### 2.2 Computer Vision–Driven Quality Assessment Bridging Human Expertise and Machine Intelligence

To replicate the master's visual evaluation throughout the tea tasting process, from observing dry tea to brewing, we integrate the master's assessment criteria with machine vision. During tea tasting, the master evaluates three core aspects: dry tea appearance, infused leaf, and tea soup. Dry tea is assessed based on flatness, straightness, smoothness, tenderness, greenness, glossiness, and color uniformity (46 features). The infused leaf is evaluated for tenderness, color, brightness, and evenness (25 features). Tea soup is judged by color, brightness, and turbidity (23 features). The master provides both qualitative comments and quantitative ratings, which are incorporated into our dataset to enable the model to perform fine‐grained evaluations at a master level. Therefore, we collected images of the appearance of dry tea leaves, the infused leaf, and the soup color as datasets for model construction.

#### 2.2.1 Human–Machine Collaborative Grading of Dry Tea Appearance via Intelligent‐Sensory Modeling

Tea appearance contributes 25% of the total evaluation score, making it crucial for consumers and merchants to assess tea. For the advantages of nondestructive, accurate, and fast, feature extraction and convolutional neural network (CNN) machine learning models are widely used for classification of tea images [22, 23]. However, the digital information such as color features, texture features, and shape features extracted by feature extraction methods based on images cannot be linked to the human evaluator's comments on the appearance of tea, so we established Intelligent‐Sensory to make the tea images match with the segmented dimensions of tea appearance such as straightness, moistness, and tenderness, and then give comments while giving the scores and rating of tea appearance. To compare the performance of the Intelligent‐Sensory, two grading approaches were developed: Intelligent‐Sensory combined visual tea images with human sensory evaluation comments, correlating model‐generated comments with scores to calculate the appearance score and Feature‐Extraction used traditional feature extraction and machine learning regression to assess tea appearance from images.

**FIGURE 2:**
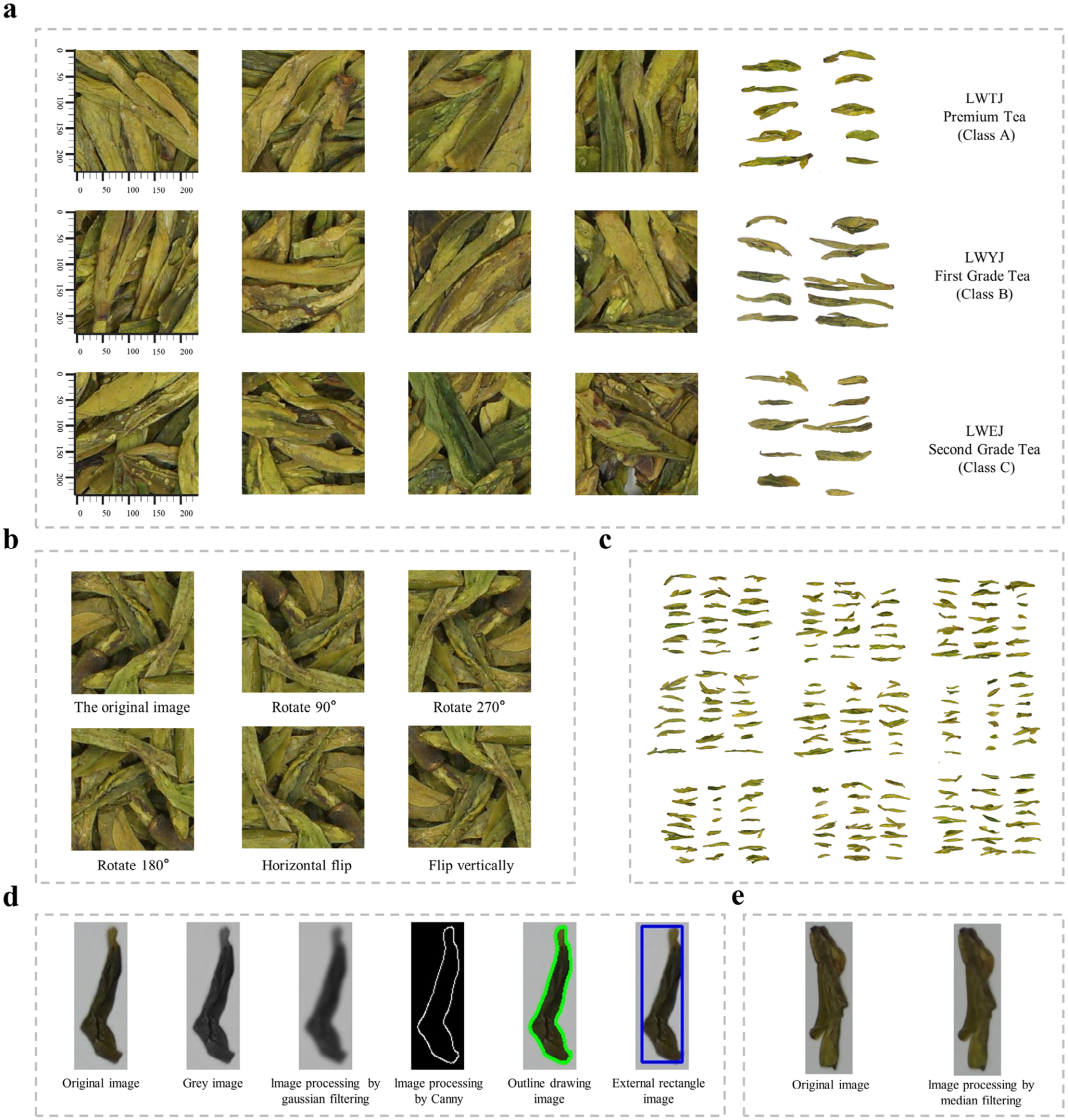
Tea appearance and image‐preprocessing pipelines. (a) Representative images of stacked and dispersed tea across three standard grades (premium, first grade, and second grade). (b) Data augmentation techniques (e.g., rotation and flipping) applied to stacked tea images. (c) Standardized arrangement for acquiring dispersed tea images. (d) Preprocessing flowchart for shape feature extraction. (e) Preprocessing flowchart for color feature extraction. (*Note*: The representative images shown are drawn from the complete vision dataset.).

Two image datasets from the same batch samples were utilized: the Intelligent‐Sensory method used stacked tea images (left part of Figure 2a), while Feature‐Extraction used dispersed tea images (Figure 2c). Both methods produced appearance scores but did not initially classify the tea. Model classification accuracy was evaluated by establishing score ranges for sensory evaluation (C < 88 ≤ B < 91 ≤ A), resulting in 12 As, 16 Bs, and 10 Cs.

#### 2.2.2 Intelligent‐Sensory

This method combined visual images with artificial sensory assessment comments. The seven‐dimensional submodel was retained for training. Tea appearance was evaluated across seven dimensions: straightness, smoothness, tenderness, greenness, glossiness, color uniformity, and integrity. The camera captured only the front side of the leaves; thereby, we omitted flatness from the evaluation. For each dimension, a submodel produced evaluation comments matched with a scoring table (Table S3). The scores for all subdimensions were summed to determine the overall appearance score. Table S1 (Sheet 2) shows the number of stacked tea images per sample, with a total number of images was 7,763. After data enhancement (Figure 2b), the amount of data has increased by six times: 75% were used for training and augmentation, and 25% for testing.

**FIGURE 3:**
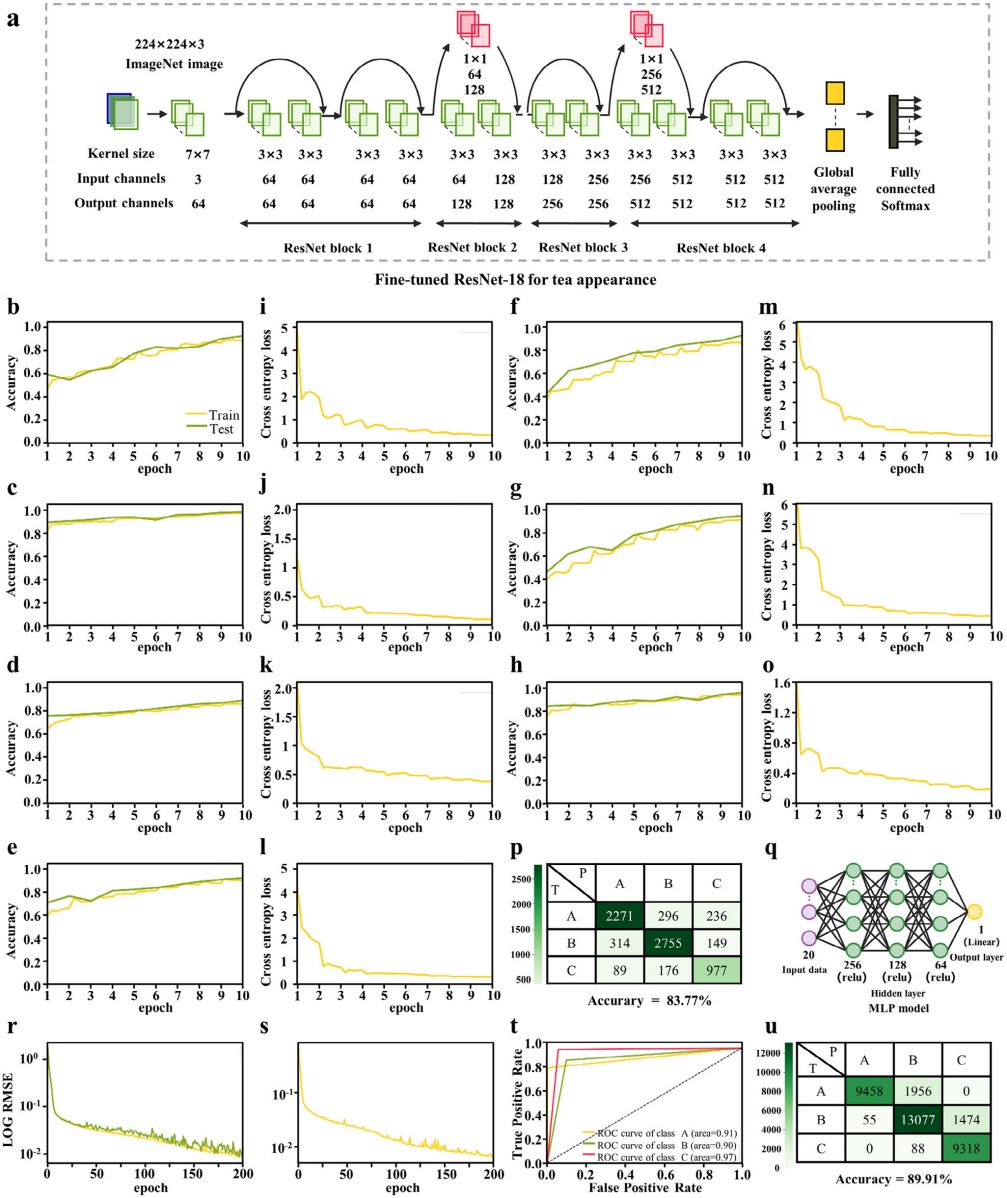
The training results of tea appearance. (a) Fine‐tuned ResNet‐18 model diagram for tea appearance. Accuracy and loss curves for fine‐tuned Resnet‐18 scoring of seven dimensions; accuracy curves: (b) smoothness, (c) green degree, (d) tenderness, (e) glossiness, (f) color uniformity, (g) integrity, and (h) straightness. Loss curves: (i) smoothness, (j) green degree, (k) tenderness, (l) glossiness, (m) color uniformity, (n) integrity, and (o) straightness. (p) Tea appearance classification confusion matrix after summing the scores of each dimension. (q) MLP model schematic for the dispersed tea dataset. (r) Training loss curve of dispersed tea image. (s) Test loss curve of dispersed tea image. (t) ROC curve of dispersed tea image. (u) Confusion matrix of dispersed tea image. (*Note*: The performance metrics in panels (p) and (t, u) were evaluated on the independent test sets with *n* = 7263 and *n* = 35,426 samples, respectively).

A fine‐tuned ResNet‐18 CNN model (Figure 3a) was used to classify tea samples based on visual images. The accuracy and loss curves for the seven dimensions (Figure 3b–o) indicate convergence after seven epochs, exhibiting high accuracy and low loss. Figure S2 and Table S4 depict the training performance for each dimension, including loss, accuracy, confusion matrix, and classification metrics. The ResNet‐18 model achieved test accuracies of >0.85 for nearly all dimensions, with the highest accuracy for greenness (0.976) and the lowest for tenderness (0.863). Appearance classification accuracy was 83.77%, as illustrated in the confusion matrix (Figure 3i) and classification metrics (Figure S3a).

#### 2.2.3 Feature‐Extraction

To obtain detailed image information, feature extraction involved three aspects: morphology [24], color, and texture [22]. Six morphological indicators were identified: leaf length, width, aspect ratio, perimeter, area, and rectangularity. Nine color indicators represented the mean R, G, B, H, S, V, L, a*, and b* values. Texture features were derived using a gray‐level co‐occurrence matrix by averaging five statistical measures (contrast, dissimilarity, homogeneity, correlation, and angular second moment) across four directions (0°, 45°, 90°, and 135°). We utilized these 20 indicators, and the values from 10 leaves were averaged to form a training sample. Table S5 and Data S1 present the detailed parameters. Images were subjected to preprocessing, including grayscale transformation and edge detection utilizing the Canny operator (Figure 2d,e).

The dataset contained five dispersed tea images of tea leaves per sample, arranged into 9 × 3 × 7 matrices (Figure 2c), totaling 945 leaves per sample. A total of 390 images represented approximately 35,426 tea single leaves, classified into class A (14,916 leaves), class B (14,977 leaves), and class C (5533 leaves). Typically, 75% of the data were used for training and 25% for testing.

Regression models utilized appearance dimension scores from sensory evaluations as labels. Random forest regression (RFR), gradient boosting regression trees (GBRT), support vector regression (SVR), XGBoost, and MLP were employed. The *r*, *R* ^2^, and LOG RMSE were utilized as evaluation metrics: the *r* represents the correlation coefficient, *R* ^2^ indicates the explanatory power, and LOG RMSE quantifies the prediction error. Fivefold cross validation was applied to enhance model robustness. Classification metrics in Figure S3f indicated that MLP (Figure 3q) achieved the highest *R* ^2^ of 0.9741 and the lowest LOG RMSE of 0.0061, which means MLP has the highest fit and was the most suitable of the five regression models for tea appearance feature extraction dataset. The MLP loss curves converged at 150 epochs (Figure 3r,s). The receiver operating characteristic (ROC) curve (Figure 3t) depicts an area under the curve (AUC) of 0.97 for class C, surpassing class A (0.91) and class B (0.90). The confusion matrix (Figure 3u) depicts a classification accuracy of 89.91%, indicating minimal misclassification (Figure S3b).

On the accuracy of the classification of the single dimension of tea appearance, the MLP model in Feature‐Extraction achieving 89.91% accuracy, surpassed the ResNet‐18 model in Intelligent‐Sensory (83.77%). However, this does not indicate the superiority of one method for comprehensive evaluation. Because appearance accounted for only 25% of the comprehensive evaluation, the classification accuracy of a single dimension cannot be directly linked to the accuracy of the comprehensive evaluation, which was related to the inconsistency of the grading intervals of the different dimensions. Thus, both Intelligent‐Sensory and Feature‐Extraction were retained for subsequent comprehensive model comparisons.

#### 2.2.4 Automated Assessment of Infused Leaf and Soup Color Through Morpho‐Colorimetric Profiling

Feature‐Extraction was applied to infused leaf and soup color assessment due to data limitations, ensuring effective evaluation of color and texture. The infused leaf and soup color contributed 10% of the tea total score, respectively. The use of the Intelligent‐Sensory requires enough data, and the number of images of infused leaf and soup color is limited. So, Feature‐Extraction is used for infused leaf and soup color model construction. Infused leaves, examined for tenderness, color, and uniformity, are assessed in a similar manner to that of dry tea appearance. Only color and texture features were extracted due to the difficulty in defining wet leaf edges. For soup color, the sensory evaluation focused on color type, chroma, brightness, and clarity, utilizing the same feature extraction method as for color feature of tea appearance.

For infused leaf classification, score ranges (C ≤ 87< B ≤ 89< A) resulted in 17 class A, 12 class B, and 9 class C samples. A similar procedure was applied for soup color (C ≤ 87< B ≤ 89< A), with 15 class A, 16 class B, and 7 class C samples. One image was obtained per sample, and dataset augmentation increased the number of images to five times. The data after feature extraction of infused leaf and soup color pictures are shown in Data S2 and S3.

**FIGURE 4:**
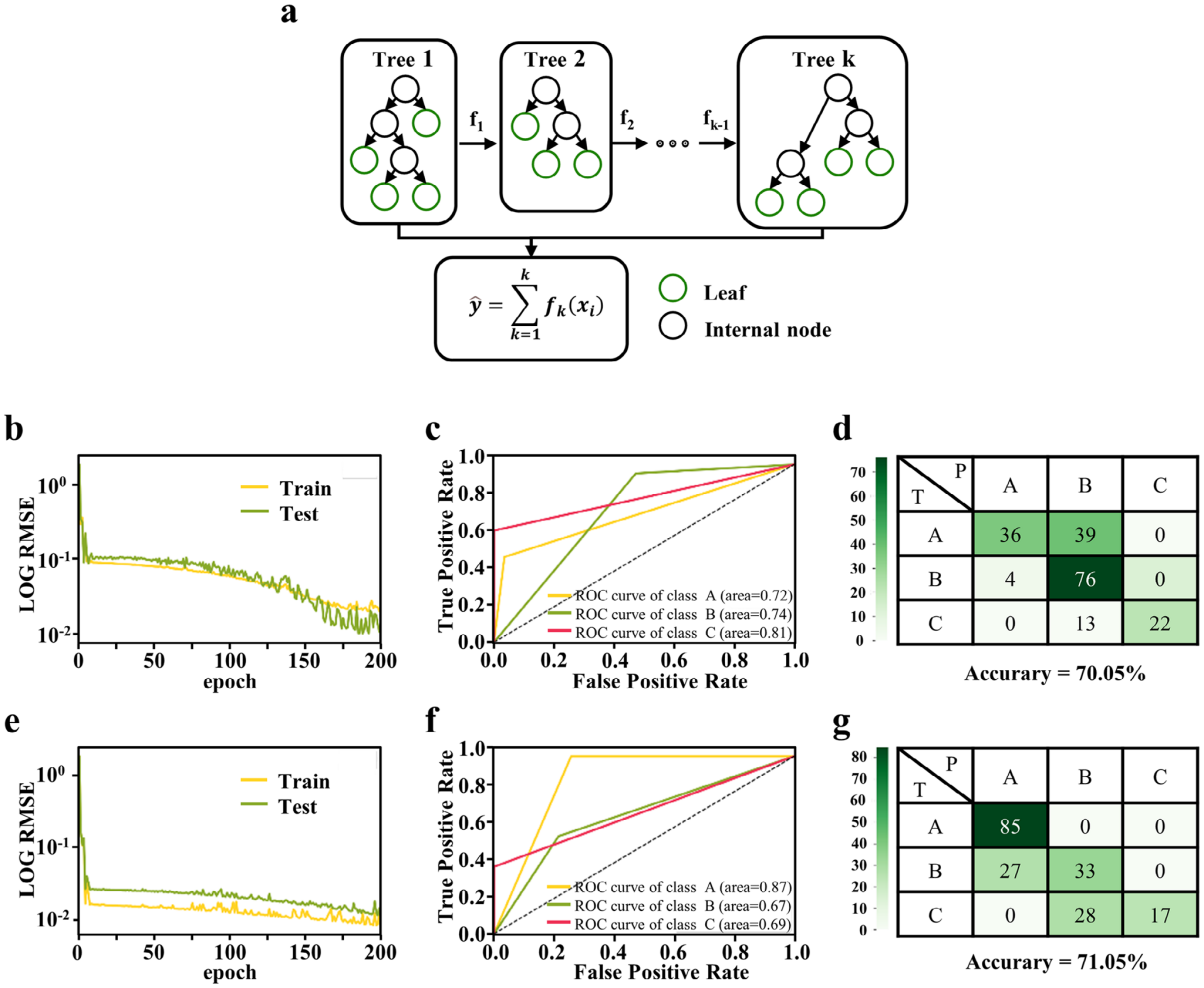
Training and evaluation results for soup color and infused leaf grading using the XGBoost model. (a) Schematic diagram of the XGBoost model. (b–d) Model performance for soup color: (b) training and test loss (Log RMSE) curves, (c) ROC curves for the three classes, and (d) confusion matrix. (e–g) Model performance for infused leaf: (e) training and test loss (Log RMSE) curves, (f) ROC curves for the three classes, and (g) confusion matrix. (*Note*: The performance metrics, including ROC curves and confusion matrices, were evaluated on the independent test sets with *n* = 190 samples for both modalities).

Five algorithms—RFR, GBRT, SVR, XGBoost, and MLP—were employed to build scoring models for both dimensions. XGBoost (Figure 4a) achieved the highest *R* ^2^ values (0.9944 for infused leaf and 0.9969 for soup color) and the lowest LOG RMSE (0.0018 for infused leaf and 0.0011 for soup color) (Figure S3g,h). The loss curves converged at 175 epochs (Figure 4b,e). ROC curves (Figure 4c,f) depicted superior classification for category C in soup color (AUC = 0.81) and category A in infused leaf (AUC = 0.87). Confusion matrices (Figure 4d,g) depict 70.05% accuracy for soup color and 71.05% for infused leaf, which were much lower than the 89.91% accuracy for tea appearance, likely attributable to insufficient data. Misclassifications predominantly occurred between classes A and B or classes C and B (Figure S3c,d).

### 2.3 Neural Network–Driven Aroma Grading From Volatile Organic Compound Landscapes

**FIGURE 5:**
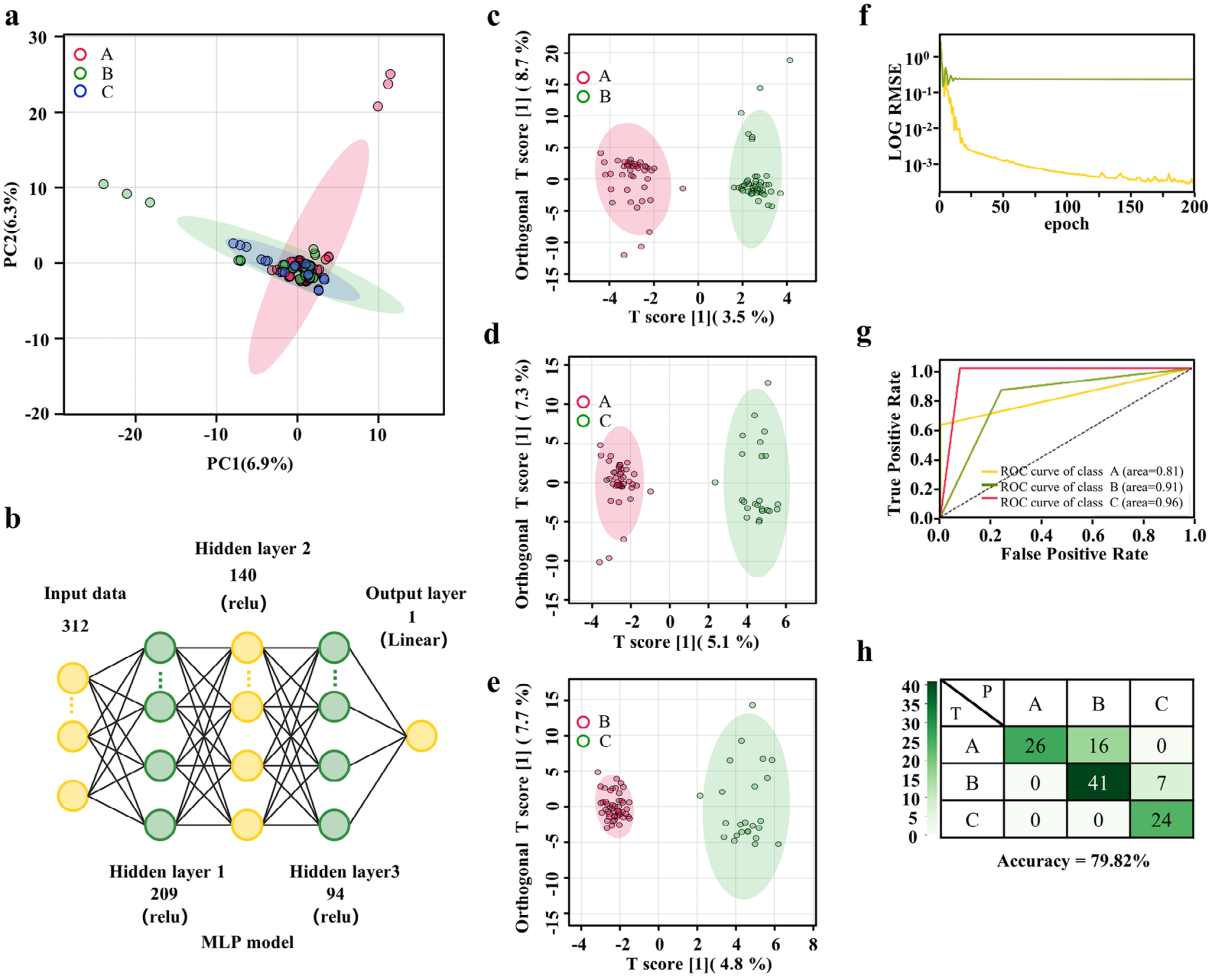
GC‐MS metabolomics analysis and MLP model performance for tea aroma grading. (a) Principal component analysis (PCA) score plot distinguishing the three tea classes. (b) Schematic architecture of the multilayer perceptron (MLP model. (c–e) Orthogonal partial least squares discriminant analysis (OPLS‐DA) score plots for pairwise comparisons: (c) class A vs. class B, (d) class A vs. class C, and (e) class B vs. class C. (f) Training and test loss (Log RMSE) curves over epochs. (g) ROC curves and (h) confusion matrix evaluating the MLP model. (*Note*: The PCA and OPLS‐DA models were constructed using tea class A, *n* = 42; tea class B, *n* = 48; and tea class C, *n* = 24. The ML performance metrics in panels (g, h) were evaluated on the independent test set with *n* = 114 samples).

The masters assess tea aroma by sniffing the infused leaf, evaluating it based on aroma type, purity, and persistence. Due to the limited data available for each sample, establishing a high‐fidelity mapping between compound data and subdimension comments was infeasible. Therefore, the ratings were directly used as the model's output. In aroma, we classified tea samples by analyzing volatile compounds in 38 Longjing tea samples (three replicates each) brewed with boiling water using solid‐phase microextraction gas chromatography–mass spectrometry (SPME‐GC‐MS). A total of 298 kinds of aroma compounds were identified, including aldehydes, ketones, alcohols, terpenes, acids, benzenes, cycloalkanes, aromatics, and amino acid derivatives, based on retention times and the NIST 20 database (Data S4). As an unsupervised statistical method, principal component analysis (PCA) was utilized for 298 variables to identify differences and correlations among variables and samples. The contribution rates of PC1 and PC2 were 6.9% and 6.3%, respectively, indicating the limited ability of PCA to distinguish tea grades based on aroma (Figure 5a). However, orthogonal partial least squares discriminant analysis (OPLS‐DA) analysis successfully differentiated tea grades (Figure 5c–e).

We utilized sensory evaluation scores for aroma as input labels and featuring the content of volatile compounds. A regression strategy was utilized to generate aroma scores, with classification intervals defined as C < 88 ≤ B < 92 ≤ A (14 As, 16 Bs, and 8 Cs).

We trained an MLP model (Figure 5b) to classify tea samples based on aroma utilizing high‐dimensional, sparse GC‐MS aroma data (114 samples and 298 metabolite features). The loss curve (Figure 5f) illustrates that the training set LOG RMSE rapidly decreased and stabilized at 10^−3^, while the testing set exhibited minimal variation, indicating potential overfitting. The ROC curve (Figure 5g) depicts an AUC of 0.96 for class C, surpassing classes A (0.81) and B (0.91). The confusion matrix (Figure 5h) indicates 79.82% accuracy, revealing significant confusion between classes A and B, while class C is classified effectively. The macro and weighted averages (Figure S3e) for precision, recall, and *F*1‐scores vary between 0.79 and 0.83. These metrics demonstrate satisfactory accuracy, with significant misclassification between classes A and B.

MLP outperformed the other models, exhibiting significant advantages in handling the high‐dimensional sparse data. Four other models (RFR, GBRT, SVR, and XGBoost) were created to predict aroma scores (Figure S3i) in addition to MLP. The *R* ^2^ scores demonstrated that MLP achieved the best performance at 0.9998, followed by XGBoost (0.9964) and SVR (0.9949). RFR and GBRT exhibited *R* ^2^ scores of 0.9712 and 0.9840, respectively.

### 2.4 Neural Network Taste Classification Optimized With Metabolomic Biomarker Screening

The master's evaluation of tea taste is broken down into four aspects: purity, freshness, density, and coordination degree. Due to the limited amount of data for each sample, we used the score as the direct output of the model. An untargeted metabolomics approach utilizing liquid chromatography–mass spectrometry was employed to digitize sensory evaluation and analyze taste compounds in tea infusion. Metabolomics is widely used to analyze metabolic profiles and correlate chemical composition with sensory qualities in tea [25, 26]. Herein, metabolic profiles were generated from 38 tea samples. After removing bias and normalizing missing values, 12,287 ions in positive mode and 9648 ions in negative mode were retained (Data S5). We identified 718 metabolites in positive mode and 265 in negative mode as candidate differential metabolites (Data S6). PCA and OPLS‐DA were employed to assess these metabolites

OPLS‐DA effectively distinguished tea grades, outperforming PCA in clustering, while differential compound analysis highlights significant chemical variations among classes. PCA revealed that the first and second principal components accounted for 21.5% and 11.9% of the variance, respectively, but did not effectively differentiate the tea grades (Figure S4b). OPLS‐DA demonstrated good clustering (Figure S4c–e), effectively distinguishing class C from classes B and A. The permutation test results validated the OPLS‐DA model, with *R* ^2^ = 0.838 and *Q* ^2^ = 0.377 for B versus C, and *R* ^2^ = 0.721 and *Q* ^2^ = 0.356 for A versus C (Figure S4i–k). Volcano plot analysis (Figure S4f–h) revealed 124 differential compounds (VIP > 1, 64 upregulated and 60 downregulated) between classes B and C and 147 differential compounds (VIP > 1, 51 upregulated and 96 downregulated) between classes A and C (Figure S4f–h), demonstrating significant differences between these groups.

Metabolite profiling revealed distinct chemical differences among tea classes, with sugars contributing to superior flavor. Figure S5a,b exhibits variations in 10 major metabolites and 18 metabolic biomarkers across the three tea classes. Sugars like sucrose and trehalose concentrations were significantly lower in class C than in classes A and B, with class A exhibiting the highest levels. The higher concentration of these sugars in class A may enhance its superior flavor profile. Additionally, compounds including 3‐hydroxy‐5Z‐octenyl acetate and Aegle marmelos alkaloid C were higher in class C than in the other classes (Figure S5c). However, chemical compounds [27, 28] including flavonoids (catechins and gallic acid), alkaloids (caffeine), and amino acids (theanine) are typically used in tea grading. No significant differences were observed for these substances among the three tea grades (Figure S5d).

**FIGURE 6:**
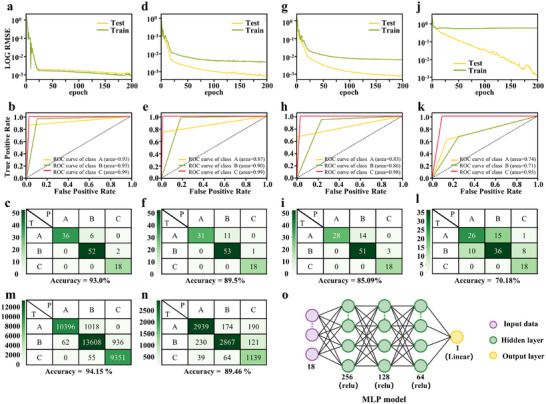
Performance evaluation of different taste datasets and the overall multimodal fusion framework. (a–c) DATA_MS2, (d–f) DATA_ANOVA, (g–i) DATA_OPLS‐DA, and (j–l) DATA_PCA: evaluation results including training/test loss (Log RMSE) curves, three class ROC curves, and confusion matrices. (m) Five‐dimensional weighted result confusion matrix—appearance: Feature‐Extraction + MLP; soup color and infused leaf: XGBoost; and aroma and taste: MLP. (n) Five‐dimensional weighted result confusion matrix—appearance: Intelligent‐Sensory + ResNet; soup color and infused leaf: XGBoost; and aroma and taste: MLP. (o) MLP model schematic for taste datasets. (*Note*: All performance metrics in panels (a–l) were evaluated on the test set with *n* = 114 samples per modality (class A: 42, class B: 48, and class C: 24). The fusion results in panels (m, n) were derived from the aggregated multimodal framework).

Then, a regression strategy was employed to classify tea samples based on taste. Score intervals (C < 88 ≤ B < 91 ≤ A) were defined, yielding taste‐dimension classifications (14 As, 16 Bs, and 8 Cs). An artificial neural network (ANN) model utilizing MLP (Figure 6o) was implemented, employing a metabolomics database comprising 38 tea samples containing 983 identified compounds derived from secondary mass spectrometry. Initially, the utilization of 100 metabolites led to overfitting and 30% accuracy on the test dataset. Minimizing the input to 18 metabolites enhanced model accuracy to 99%. Four strategies were utilized to identify these 18 metabolites: (1) Data_analysis of variance (ANOVA): selection of the 18 metabolites with minimum *p*‐values based on ANOVA analysis of the ABC classes; (2) Data_MS2: selection of top 18 metabolites with the highest secondary mass spectrometry scores; (3) Data_PCA: selection of the first 18 principal components from PCA analysis; (4) Data_OPLS‐DA: identification of differential metabolites with VIP > 1, intersection of two‐by‐two analyses in three ABC classes via OPLS‐DA. This process yielded ten candidate metabolites, which is fewer than expected. Table S6 presents these four datasets.

The MLP was applied to the datasets filtered by each of the four strategies. The training and testing loss curves (Figure 6a,d,g,j) demonstrate the performance of the model. Initially, the LOG RMSE for training and testing sets diminished rapidly, indicating rapid acquisition of key features. ROC curves (Figure 6b,e h,k) evaluated model performance, revealing the AUC for classes A, B, and C in the MS2 dataset being 0.93, 0.93, and 0.99, outperforming other datasets. Confusion matrices (Figure 6c,f,i,l) revealed the highest total accuracy of the MS2 dataset (93.0%), outperforming ANOVA (89.5%), OPLS‐DA (85.09%), and PCA (70.18%). Other classification metrics, including precision, recall, and *F*1‐score, are illustrated in Figure S6a–d. The *r*, *R* ^2^, and LOG RMSE values for the MLP model across datasets are presented in Figure S6e. The *R* ^2^ score, an essential performance metric, was high for MS2, ANOVA, and OPLS‐DA datasets (0.9982, 0.9970, and 0.9992, respectively), surpassing PCA (0.9783). Despite the lower classification accuracy than some datasets, the OPLS‐DA dataset was selected for the taste dimension due to its strong regression fit.

Four other models, RFR, GBRT, SVR, and XGBoost, were developed to predict taste scores in addition to the MLP model. MLP attained the highest *R* ^2^ values in most datasets (Table S7). MLP demonstrated superior fitting ability and prediction accuracy, making it the optimal choice for the taste dimension.

### 2.5 Multimodal AI System for Comprehensive Quality Prediction

In the final part of the manual evaluation, the total score of the tea needs to be obtained by weighting and summing up the scores of the five dimensions. Before calculating the total score, the master evaluator will also go back to verify the results of the previous evaluation and make secondary adjustments to correct bias. We applied the multimodal algorithm Tip‐CLIP as a supervisor in Long‐Tea to exercise this bias correction function.

#### 2.5.1 Weighted Fusion of Multisensory Modalities

After creating assessment models for tea appearance, aroma, taste, soup color, and infused leaf, the method with the highest fit was selected as the submodel for the comprehensive classification. Each submodel generates scores for its dimension, which are utilized to calculate the final overall score. The MLP model was selected for aroma and taste, while XGBoost was selected for infused leaf and soup color. Tea appearance was assessed respectively using Intelligent‐Sensory and Feature‐Extraction: one involving stacked tea and another with dispersed tea. The Intelligent‐Sensory method utilized fine‐tuned Resnet‐18 deep‐learning visual models for appearance scores, while the Feature‐Extraction method employed MLP. Utilizing different datasets, both methods function as submodels within the appearance dimension to determine the final tea scores. To solve the problem of inconsistent data volumes across dimension datasets and objectively evaluate the accuracy and efficacy of the methods in practical applications, soup color, infused leaf, aroma, and taste data were randomly paired with corresponding appearance image data for each tea category with equal probability. This ensured that each image was paired with the aroma and taste data for accurate assessment.

We confirmed the effectiveness of a multimodal system for accurate tea grade classification, leveraging different assessment methods for optimal performance. The predicted scores for each assessment dimension—appearance (25%), soup color (10%), aroma (25%), taste (30%), and infused leaf (10%)—were weighted and summed to calculate the overall tea score. Tea grades were classified as C < 88 ≤ B < 90 ≤ A based on the total score. The optimal models and data‐preprocessing methods were selected for soup color, aroma, taste, and infused leaf, while an additional comparison is required for the appearance dimension. The two multimodal systems are designated Long‐Tea‐Intelligent and Long‐Tea‐Feature, respectively. Employing the dispersed tea image method (MLP), Long‐Tea‐Feature achieved 94.15% accuracy (Figure 6m). Using the stacked image method (fine‐tuned ResNet‐18) for the appearance dimension, Long‐Tea‐Intelligent achieved an accuracy of 89.46% (Figure 6n). The Feature‐Extraction for dispersed tea images yielded higher accuracy.

In addition to the weighted model modalities, we employed the more mainstream multimodal feature‐level fusion [29, 30, 31] approach (the model structure is shown in Figure S7a) for modeling. This model completed fitting in approximately 150 epochs (Figure S7b). The three class ROC plot, confusion matrix, and classification result metrics are shown in the Figure S7b–d. We found that the overall accuracy of this feature fusion model was 87.99%, significantly lower than the 89.46% and 94.15% achieved by the weighted model. Simply feeding data to the model may not achieve excellent classification performance.

#### 2.5.2 CLIP‐Supervised Multimodal Feature Fusion

**FIGURE 7:**
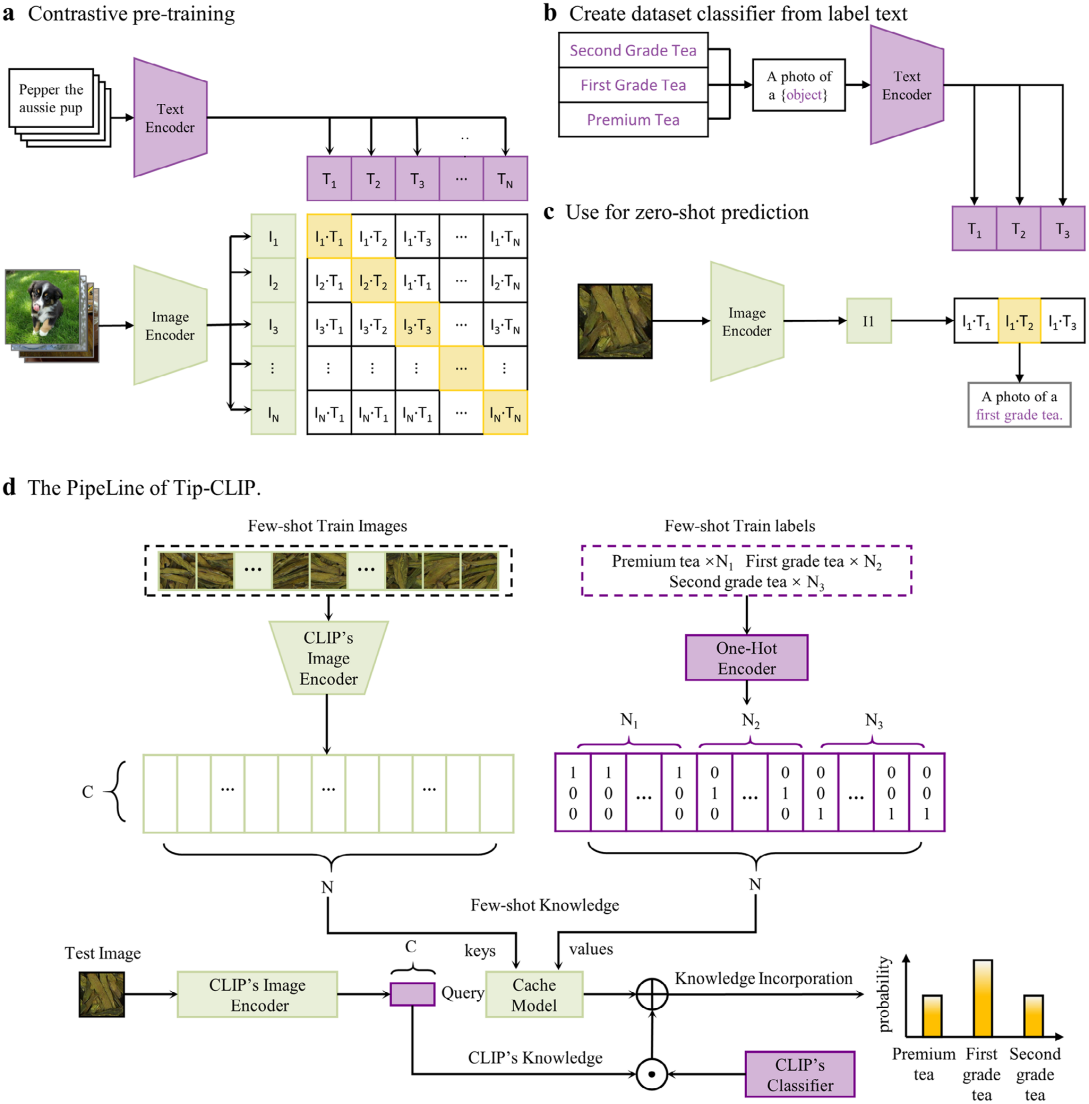
Summary of CLIP supervisor: (a) contrastive pretraining, (b) creation of dataset classifier from label text, (c) use for zero‐shot prediction, and (d) the pipeline of Tip‐CLIP. Given a few‐shot training set, the method constructs the cache model using the image encoder of CLIP to transform images as keys or queries and using the one‐hot encoder to transform labels as values. The cosine similarity between keys and queries is used as the weight for the sum of values, which represents the few‐shot knowledge.

The CLIP [21, 32] model leverages large‐scale pretraining for zero‐shot tea classification, but its general training data limited accuracy in assessing tea quality levels. The CLIP model was employed to train on pre‐existing text (soup color, infused leaf, taste, and aroma) and image (tea‐stacked images) datasets. The CLIP base model pretrained on a large‐scale (400 million) text‐image pair dataset can be transferred directly to similar datasets, such as ImageNet tasks for zero‐shot classification without requiring fine‐tuning with labeled images (Figure 7a–c). However, its pretraining on general data may not have acquired specialized representations for tea quality levels, resulting in diminished classification accuracy.

A few‐shot image classification method, Tip‐CLIP, was introduced to enhance the transfer performance of CLIP while addressing time and space constraints. This method improved the accuracy of CLIP without additional downstream training. Tip‐CLIP employs CLIP to create a cache model (Figure 7d) in a nontraining approach to retain classification knowledge derived from downstream training data (especially about tea quality grading). During testing, the classification results are derived from the linear combination of the predictions from the cache and the CLIP base model, yielding more reliable classification outcomes.

**FIGURE 8:**
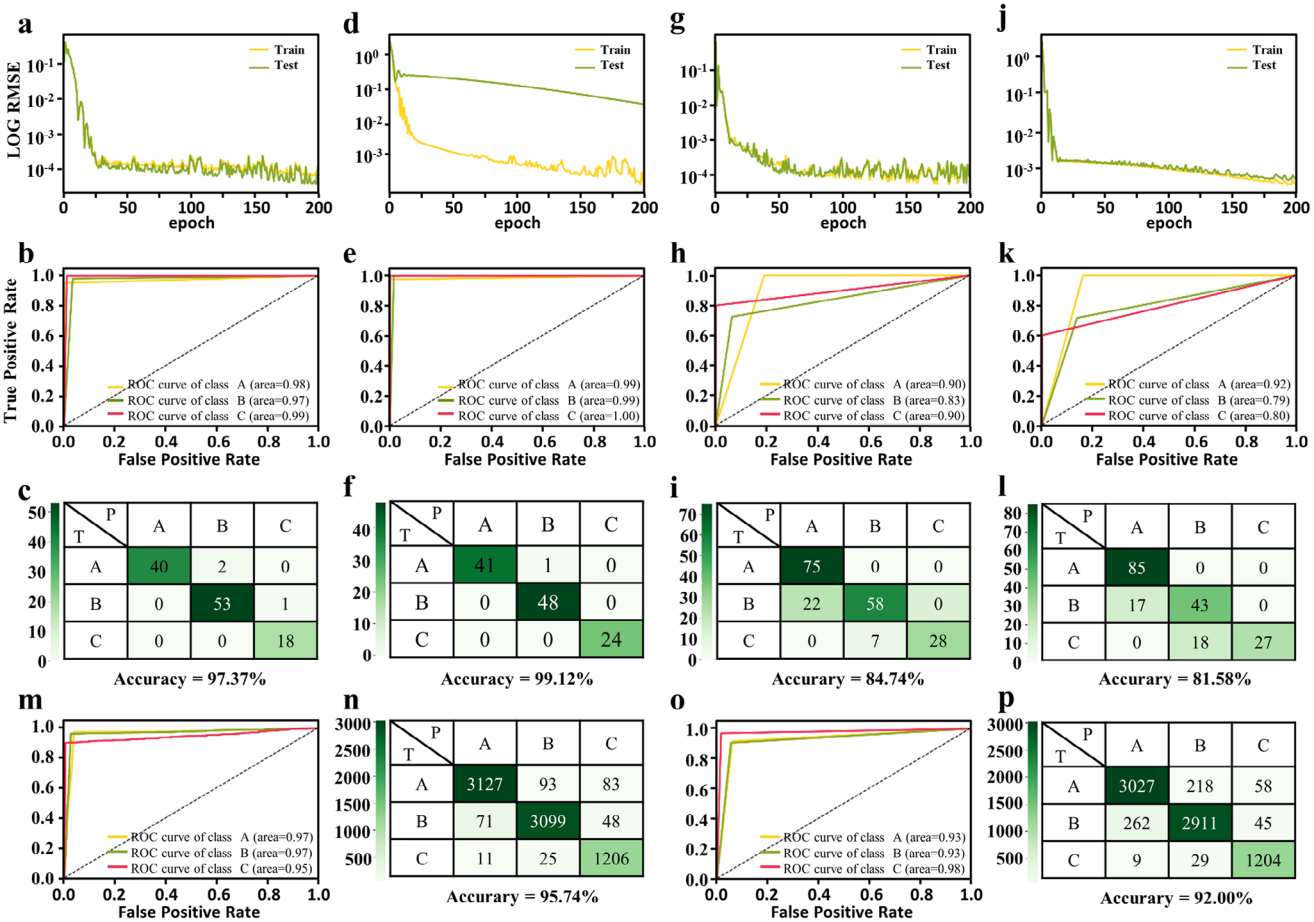
Performance of the CLIP‐based supervised training framework. (a–c) Taste, (d–f) aroma, (g–i) soup color, and (j–l) infused leaf dataset evaluation results: training/test loss (Log RMSE) curves, three‐class ROC curves, and confusion matrices. (m,n) CLIP‐based classification results using tea stacking images (as image data) and taste, aroma, soup color, and infused leaf (as text data). (o,p) Weighted classification results for the five data dimensions, integrated via ResNet (for stacking images), TCSMLP (for taste, aroma, and infused leaf), and XGBoost (for soup color). (*Note*: Performance metrics in panels (a–l) were evaluated on test sets with *n* = 114, 114, 190, and 190 samples, respectively; results in panels (m–p) were based on *n* = 7763 samples).

Enhancing the CLIP model with tea grading knowledge significantly improved classification accuracy and regression performance across multiple tea quality assessment datasets. The three‐class ROC curve and confusion matrix for CLIP (Figure 8m,n) indicate an accuracy of 95.74%, illustrating its strong classification ability for various tea grades. After enhancing the CLIP model with tea grading knowledge, the refined Tip‐CLIP model supervised regression tasks for taste, aroma, soup color, and infused leaf datasets. Figure S3g–i and Figure 5g depict the *r*, *R* ^2^, and LOG RMSE values for the four datasets. The Tip‐Clip supervised multilayer perceptron (TCSMLP), developed under CLIP supervision, significantly improved fitting performance, with *R* ^2^ values being higher than the original MLP model. Except for soup color, TCSMLP attained the highest *R* ^2^ for the infused leaf, aroma (Figure S3g–i), and taste datasets, with the MS2 dataset illustrating the highest accuracy (*R* ^2^ = 0.9999; Figure S5f). XGBoost was selected for the soup color dataset, while TCSMLP was utilized for the infused leaf, aroma, and taste datasets, with the MS2 dataset trained using TCSMLP. After training the entire dataset, the training loss curve, three‐class ROC curve, and confusion matrix were obtained (Figure 8a–c for taste, Figure 8d–f for aroma, Figure 8g–i for soup color, and Figure 8j–l for infused leaf). Table S8 presents the classification metrics. As *R* ^2^ increased, the classification accuracy of TCSMLP improved: 97.37% for taste, 99.12% for aroma, 84.74% for soup color, and 81.58% for infused leaves.

Integrating CLIP supervision into the Long‐Tea‐Clip model significantly improved classification accuracy, optimizing comprehensive tea quality assessment. ResNet was utilized to train the appearance dimension, while TCSMLP was employed for taste (MS2), aroma, and infused leaf datasets. XGBoost was employed for the soup color dataset because of its superior performance. The optimized method, Long‐Tea‐Clip, demonstrated a classification accuracy of 92.00%, significantly higher than the 89.46% achieved without CLIP supervision. ROC curves and confusion matrices (Figure 8o,p) depict that CLIP‐supervised training effectively enhanced the accuracy of Long‐Tea Intelligent.

## 3 Discussion

To develop an AI‐assisted system for objective and comprehensive tea quality grading, we developed Long‐Tea‐CLIP, a multimodal AI system that emulates the comprehensive approach and evaluation fineness of expert tea evaluators to evaluate soup color, infused leaves, appearance, aroma, and taste, achieving 92.00% classification accuracy. We have developed a comprehensive framework for tea quality assessment by digitizing visual characteristics of dry tea, tea soup, and infused tea through computer vision and analyzing aroma and taste profiles using GC‐MS and LC‐MS.

In the early stages, the AI‐driven classification of tea focused on a single dimension [24]. With the exploration of AI techniques such as multimodality and data fusion, researches on the multidimensional classification of tea based on appearance, taste, and aroma have emerged [16, 33, 34]. For instance, Ren et al. used near‐infrared spectroscopy, E‐eye, E‐tongue, and E‐nose technologies to capture the internal and external characteristics of tea. They combined data fusion with a CNN classification model, achieving a comprehensive evaluation of tea leaves with a misclassification rate of 0.86% [35]. This approach neglects two dimensions of manual sensory evaluation: soup color and infused leaf, necessitating the brewing of tea and the separation of the broth from the wet leaves. Given the extensive data requirements for machine learning, collecting data on soup color and infused leaves is more difficult than photographing dry tea. These two dimensions, comprising 20% of the sensory evaluation in GB/T 23776‐2018, are essential to the commodity value of tea. Therefore, we have taken this into consideration, and while ensuring the appearance, aroma, and flavor dimensions, we have also included soup color and infused leaf in the Long‐Tea‐CLIP.

We chose the more informative way of collecting information on tea quality. For appearance, traditional computer vision combined with feature extraction and machine learning for tea quality classification, spectral imaging technologies are increasingly being used [22, 36, 37]. In order to obtain images with richer details, we used a high‐resolution DSLR camera to take pictures. In feature extraction, considering the flat and straight appearance of green tea, we also performed shape feature extraction in addition to color and texture features. Due to the similarity of the evaluation method, the soup color and leaf bottom can also refer to the appearance of the method of data collection. For aroma and taste, addition to metabolomics supported LC‐MS [26], and GC‐MS [25, 26, 38], electronic nose [39, 40], and electronic tongue [41, 42], which are capable of nondestructive detection, have also appeared in the method of collecting data of tea aroma and taste. They are convenient to use, but still LC‐MS and GC‐MS are better in terms of breadth and depth of compound data collection. Artificial intelligence algorithms also have a better potential of exposing the relationship between compound content and tea quality in large amounts of compound data, and even elucidating the specific link between compound content and flavor.

By integrating multimodal AI supervision with traditional machine learning, Long‐Tea‐CLIP enhances tea grading accuracy, interpretability, and scalability, offering a novel, data‐driven approach to quality assessment. We utilized samples from 38 varieties of Longjing green tea, graded by internationally expert tea masters, to train AI models, including ResNet, MLP, and XGBoost. This study presents a more comprehensive evaluation of visual dimensions based on the experience of master tea tasters. High‐resolution imaging captures 30 critical quality indicators, including 7 appearance traits (straightness, smoothness, tenderness, greenness, glossiness, color uniformity, and integrity), 9 soup color parameters, and 14 infused leaf characteristics, processed through ResNet‐18 and morpho‐colorimetric algorithms. Furthermore, the training was optimized by employing the multimodal CLIP model as a supervisor to replicate expert assessment. Long‐Tea‐CLIP combines the superior performance of multimodal CLIP in few‐shot learning with the high interpretability of traditional machine learning ensemble methods. Tip‐CLIP, the semi‐supervised approach enhances conventional machine learning techniques, increasing performance from 89.46% to 92.00% while preserving effectiveness and interpretability. It helps address several limitations of traditional sensory evaluation by providing a scalable, objective, and reproducible method for assessing tea quality. Our study demonstrates the potential of combining advanced technologies with traditional expertise, resulting in enhanced quality control and consistency within the tea industry through innovative digital solutions.

In achieving this comprehensive evaluation, the proposed framework intentionally adopts a modular architecture rather than a unified end‐to‐end multitask learning (MTL) framework. This modular design is primarily motivated by the traditional sensory procedure, where each dimension reflects a relatively independent perceptual process. Although an end‐to‐end MTL framework with a shared backbone might seem theoretically appealing, such architectures may introduce task interference (e.g., negative transfer) due to the highly heterogeneous nature of tea sensory attributes and their distinct optimal feature representations. Furthermore, an underappreciated risk in MTL with highly heterogeneous data is the potential for “shortcut learning” or spurious correlations, where an end‐to‐end model might leverage a dominant signal from one modality (e.g., visual features) to predict labels for all tasks, ignoring critical but subtle chemical profiles. By physically separating the networks, our modular approach enforces that each predictor must rely on the features most relevant to its specific task. While an end‐to‐end MTL model resembles an intuitive process (fast, parallel, but opaque), our modular design aligns more closely with the step‐by‐step analytical reasoning of professional tasters, thereby significantly enhancing interpretability and robustness.

In practical deployment, however, the performance of Long‐Tea‐CLIP will depend not only on model accuracy but also on the strict standardization of multimodal data acquisition. In this study, images were collected under tightly controlled conditions (fixed lighting, camera settings, and background), and brewing conditions were rigorously standardized. Variations in these procedures in a real‐world tea factory could introduce significant nonquality‐related noise. Furthermore, although LC‐MS and GC‐MS provide rich chemical profiles, their high equipment cost and operational complexity may limit routine deployment. Therefore, different industrial scenarios require tiered system configurations: the full multimodal framework is highly suitable for premium tea evaluation or quality arbitration settings, whereas a simplified visual‐only module may be more practical for rapid, on‐site screening. Before broader implementation, cross‐device calibration and cross‐laboratory validation will be essential. In this sense, Long‐Tea‐CLIP is currently better viewed as a robust AI‐assisted decision‐support tool rather than as a complete replacement for human experts.

Beyond these deployment‐related considerations, the current model also has specific methodological limitations. First, the Intelligent‐Sensory, which integrates tea appearance feature comments, exhibits less accuracy than the Feature‐Extraction in tea appearance grading, contrary to expectations. This discrepancy may stem from the relatively large quantity of single leaf images utilized in the Feature‐Extraction, which are less informative than those in the Intelligent‐Sensory, increasing the likelihood of false negatives. Another possible reason for the underperformance of Intelligent‐Sensory is instability in the scoring output of the model. The Intelligent‐Sensory initially trains submodels on appearance images across seven evaluation dimensions to generate tea leaf scores, producing corresponding evaluation categories for each dimension. The category vectors are subsequently converted into scores utilizing an evaluation score database. Errors may occur during the categorization of the submodel and the subsequent score conversion. The accumulation of these errors may lead to instability in the model's scoring output for stacked tea pictures. Future research could investigate methods to directly map tea appearance images to scores to mitigate this instability while simultaneously generating evaluative language to ensure interpretability. Long‐Tea‐CLIP must enhance its ability to convert sensory evaluations across dimensions beyond appearance. Nevertheless, Long‐Tea has not yet established a connection between substance content data and sensory comments in the taste and aroma dimensions. Future research aims to develop a system that provides tea grading results and generates sensory evaluations for taste and aroma, offering deeper insights into the specific flavor profiles of the tea under evaluation.

Finally, the generalization ability of this AI solution must be discussed in terms of both the specific model and the universal framework. Given that green tea accounts for over 50% of global tea production, achieving expert‐level AI grading within this category holds paramount industrial significance. The current model weights were specifically optimized using 38 types of Longjing green tea. While this ensures high fidelity within this context, the specific model cannot transfer directly to black or oolong tea without further adaptation. However, the proposed five‐dimensional evaluation methodology (appearance, soup color, aroma, infused leaf, and taste) represents the universal core of all tea evaluation. Therefore, while the current dataset is specific, the overarching multimodal framework is highly generalizable. To achieve cross‐category grading in the future, this framework can be readily extended by training on new datasets using transfer learning or domain adaptation strategies.

In conclusion, integrating advanced imaging, biochemical analysis, and machine learning technologies into tea quality grading demonstrates great promise for the tea industry. Although the present study was developed for Longjing green tea under standardized conditions, it provides a solid foundation for objective quality assessment. With continued external validation, protocol standardization, and category‐specific adaptation, such AI‐assisted systems will significantly contribute to more consistent and transparent quality control in the modern tea industry. As technology advances, these innovations will enhance quality control of market transparency and facilitate the development of the tea industry.

## 4 Methods

### 4.1 Tea Samples

We collected 38 types of Longjing green tea, each with three replications. These were sourced from five vendors in Hangzhou, Zhejiang Province. Table S1 presents the details of prices and sources. The tea samples were classified into three different quality grades based on artificial sensory evaluation, with detailed information provided in next paragraph. All samples were bought directly from the retail market, packed in aluminum foil–sealed bags, and stored at 4°C before analysis.

### 4.2 Tea Sensory Evaluation Grading

Tea experts performed sensory evaluations in accordance with the green tea quality standards specified in methodology for sensory evaluation of tea, National Standard of the People's Republic of China GB/T 23776‐2018 [43]. They evaluated appearance, aroma, flavor, color, and infused leaf characteristics and provided a total score based on a weighted calculation. Each sample (3 g) was brewed in 150 mL of freshly boiled water for 4 min and subsequently evaluated for the following attributes: appearance (25%), soup color (10%), aroma (25%), taste (30%), and infused leaf (10%). Based on the West Lake Longjing group standard TXHLJ001‐2021 [44] and expert recommendations, three tea samples (LWYJ, LWEJ, and LWTJ) were utilized as standards.

### 4.3 Image Collection and Preprocessing

#### 4.3.1 Stacked Tea Image Shooting

A custom‐built computer vision system (Figure S1a) captured images of the tea samples. The system was enclosed in black opaque acrylic sheets to replicate a darkroom, with a Sony ILCE‐7M2 digital single‐lens reflex camera fitted with a 50 mm prime lens positioned 350 mm above the samples. Three light‐emitting diode (LED) strips provided stable white illumination. For each sample, 5 g of tea was placed on a 400 × 400 mm platform. The raw images were saved in JPEG format utilizing RGB color mode. All images were taken with consistent camera settings: M mode, aperture *f*/4.5, shutter speed 1/160, ISO 100, automatic white balance, 0 exposure compensation, wide‐area focus, single‐focus mode, and multi/center metering mode. Each sample generated 200 images [22].

The raw images possessed a resolution of 6000 × 4000 pixels. In contrast to typical fruit or vegetable images, with clear boundaries between objects and their background, tea images comprised stacked leaves, making exact segmentation unnecessary. Image preprocessing involved extracting a 224 × 224 pixel region of interest (ROI) by centering the raw image, as illustrated in left part of Figure 2a.

Visual inspection of Figure 2a reveals that tea images of different quality exhibit visual similarity, presenting challenges for image‐based classification algorithms. High‐quality tea leaves typically exhibit a full, consistent shape and color with minimal impurities, whereas low‐quality leaves are frequently curled, shriveled, and discolored, with visible impurities, including broken leaves.

Approximately 200 images were collected per sample, with the dataset being divided into 75% for training and validation and 25% for testing. Data augmentation techniques, including image rotations (90°, 180°, and 270°) and flips (vertical and horizontal), were applied to images to enhance the dataset, as illustrated in Figure 2b. This procedure increased the dataset size sixfold, enhancing model generalization and mitigating overfitting [45]. Table S1 presents comprehensive details regarding the tea image datasets and data augmentation methods.

#### 4.3.2 Dispersed Tea Leaf Shooting and Preprocessing

Photographing stacked tea leaves alone does not capture the shape of individual leaves accurately, as the overlap conceals their edges. Additionally, performed dispersed tea leaf photography to obtain a more detailed understanding of tea leaf shape. All parameters remained constant except for the lens, which was replaced with a 28–70 mm zoom lens adjusted to a focal length of 30 mm. The camera was situated 600 mm from the tea leaves, utilizing A3 white paper as the background on the sample platform. Each dispersed tea image was arranged into a 3 × 3 grid, dividing the layout into three equal parts horizontally and vertically. This layout was consistent across all samples, each comprising 9 × 3 × 7 matrices, as illustrated in Figure 2c. We captured five images per sample, resulting in 945 individual data points for each sample. In total, 38 samples were photographed, generating 390 images, and capturing approximately 35,426 individual Longjing tea leaves. Of these data, 75% was utilized for training and 25% for testing [24].

Each of the 38 tea types was arranged with 9 × 7 × 3 leaves uniformly placed under the lens, all aligned horizontally to improve the efficiency of image acquisition for the dispersed tea dataset. Five images were captured for each tea type, forming the original dataset. Recognition and extraction processes were implemented on each image to isolate individual tea leaf features. The image was initially converted to grayscale, followed by the application of Gaussian filtering to reduce noise. Leaf edges were subsequently detected using the Canny operator, converting the image to a binary format. Based on these results, OpenCV was employed to extract feature parameters, including leaf length, width, aspect ratio, perimeter, area, and rectangularity. This rectangle was utilized for image cropping and rotation. For shape feature extraction, the process included grayscale conversion, Gaussian noise reduction, edge binarization, and the calculation of the minimum bounding rectangle and contour area (Figure 2d). For color feature extraction, the cvtColor function from OpenCV was utilized to convert the image to color spaces including R, G, B, H, S, V, L, a*, and b* values, while the split function calculated color features based on pixel values. For morphological feature extraction, the image was initially converted to grayscale, and grayscale levels (0–255) were mapped to 16 levels to diminish the dimensionality of the grayscale covariance matrix. Morphological features (contrast, dissimilarity, homogeneity, correlation, and angular second moment) were subsequently calculated from the covariance matrix utilizing four directional offsets.

#### 4.3.3 Infused Leaf and Soup Color Image Shooting

Before photographing the infused leaf and soup color, we followed the methodology specified in GB/T 23776‐2018 for tea sensory evaluation. The infused leaf was separated from the tea broth before photography. The soup color was assessed following the initial infusion. The 38 tea samples were divided into three batches, each containing 12–13 samples. For each sample, 3 g of tea was placed in an evaluation cup and infused in 150 mL of boiling water for 4 min, covered with a lid. The tea broth was subsequently drained into an evaluation bowl and allowed to cool for 25 min, until no water vapor was observable. The broth was placed in a white porcelain bowl to inhibit light transmission, and images were captured using the photography system. Because of the limited samples, only one image of the soup color was taken per sample, yielding 38 images. Similarly, the infused leaf was placed in a white porcelain bowl, and the photographic parameters corresponded to those used for photographing stacked tea leaf shapes. One image of the infused leaf was taken per sample, yielding 38 images. The color features of the soup and infused leaf and texture feature extraction followed the methods used for dispersed tea leaf images.

### 4.4 Extraction and Analysis of Volatile Compounds

Volatile compounds were extracted from tea samples (0.5 g) sealed in 20 mL glass vials, to which ethyl caprate (internal standard, 10 µL, 10 mg/L) and 5 mL of boiling deionized water were added sequentially. After equilibrating for 5 min, the vial was placed in a 60°C water bath, and headspace volatiles were absorbed for 60 min using a divinylbenzene/carboxen/polydimethylsiloxane coating fiber (50/30 µm, StableFlex (2 cm), Supelco, Inc., Bellefonte, PA, USA). The volatiles were desorbed at 250°C for 5 min in the GC‐MS injector. Volatile analysis was performed using an Agilent 7890 gas chromatograph coupled to an Agilent HP 5977 MSD ion trap mass spectrometer (Wilmington, DE, USA), equipped with an HP‐5MS capillary column (30 m × 250 µm × 0.25 µm). GC conditions were as follows: an inlet temperature of250°C and high‐purity helium (99.999%) utilized as carrier gas at a flow rate of 1.0 mL/min (split less mode). The temperature program comprises 40°C for 2 min, increased to 85°C at 2°C/min, maintained for 2 min; increased to 180°C at 2.5°C/min, maintained for 2 min; increased to 230°C at 10°C/min, maintained for 2 min. Mass spectrometry was performed at 70 eV in electron ionization (EI)ultra‐high‐performance liquid chromatography mode, with a mass scan range of 40–400 *m*/*z* and an ion source temperature of 230°C [46].

The NIST mass spectral search program (NIST library 20) and an internally developed GC‐MS data analysis program based on Microsoft Excel were utilized to identify volatile compounds. Identification of GC peaks was achieved by matching mass spectra and retention indices. Semiquantitative concentrations of volatiles were calculated by comparing the MS total ion current response to that of the internal standard.

### 4.5 Tea Soup Metabolomics Analysis by LC‐MS

#### 4.5.1 Metabolite Extraction

For metabolite extraction, 1 g of sample was mixed with 50 g of boiling water, thoroughly agitated, and heated in a 100°C water bath for 30 min. The mixture was shaken every 10 min, with two repetitions. The sample was subsequently centrifuged at 8000 rpm for 10 min, and the supernatant membrane was discarded. The sample was centrifuged at 12,000 rpm (13,800 × *g*) at 4°C for 15 min. The supernatant was collected and transferred to an injection vial for analysis. For QC, an equivalent number of supernatants from each sample was pooled for machine testing.

#### 4.5.2 LC–MS/MS Analysis

LC–MS/MS analyses were performed using a ultra‐high‐performance liquid chromatography (UHPLC) system (Vanquish, Thermo Fisher Scientific) with a UPLC HSS T3 column (2.1 × 100 mm, 1.8 µm) coupled to an Orbitrap Exploris 120 mass spectrometer (Thermo Fisher Scientific). The mobile phase comprises 5 mmol/L ammonium acetate and 5 mmol/L acetic acid in water (A) and acetonitrile (B). The autosampler was maintained at 4°C, and the injection volume was 2 µL. The Orbitrap Exploris 120 mass spectrometer was operated in information‐dependent acquisition mode under the control of Xcalibur software (Thermo Fisher). The electrospray ionization (ESI) source conditions were as follows: sheath gas flow rate = 50 Arb, auxiliary gas flow rate = 15 Arb, capillary temperature = 320°C, full MS resolution = 60,000, MS/MS resolution = 15,000, collision energy = 10/30/60 in normalized collision energy (NCE) mode, and spray voltage = 3.8 kV (positive) or 3.4 kV (negative) [47].

Raw data were converted into mzXML format utilizing ProteoWizard and subsequently processed with an in‐house program based on XCMS (R language) for peak detection, extraction, alignment, and integration. Metabolite annotation was performed using an in‐house MS2 database (BiotreeDB), with an annotation cutoff of 0.3.

### 4.6 Data Processing Analysis and Algorithms

Principal component analysis is a commonly used method for dimensionality reduction. It transforms a set of correlated variables into a smaller set of uncorrelated variables, known as principal components. The initial principal component accounts for the largest variance, followed by others in decreasing order. PCA reduces data dimensionality while maintaining maximal original information as much as possible. Before PCA, data were normalized to mitigate the effects of outliers.

OPLS‐DA is a statistical method that differentiates and explains class differences in multivariate datasets. OPLS‐DA enhances the interpretive and predictive accuracy of the model by decomposing predictor variables into class‐dependent and class‐independent orthogonal components. It is widely used in metabolomics, genomics, and related fields to identify biomarkers and classify features. OPLS‐DA more effectively removes class‐independent noise than PLS‐DA, enhancing model robustness and explanatory power.

To adapt the pretrained CLIP model for the specialized task of tea grading, we developed a few‐shot learning approach named Tip Clip. This method constructs a nonparametric cache model from a small support set to inject domain‐specific knowledge at inference time. The cache consists of feature “Keys” (Equation 1) extracted from support images and corresponding one‐hot encoded “Values” (Equation 2):

(1) $$\mathrm{Key}\;=\;\mathrm{VisualEncoder}\left(I_{k}\right)\in R^{NK\times C}$$

(2) $$\mathrm{Value}\;=\;\mathrm{OneHot}\left(L_{k}\right)\in R^{NK\times N}$$

For a given test image, it is encoded into a “Query” vector (Equation 3), and the final classification “Logits” (Equation 5) are derived by combining the standard CLIP prediction with the cache model's output, weighted by the similarity (Equation 4) between the Query and the Keys:

(3) $$\mathrm{Query}\;=\;\mathrm{VisualEncoder}\left(I\right)\in R^{1\times C}$$

(4) $$\mathrm{Similarity}\;=e^{-\beta \left(1-\mathrm{Query}\times \mathrm{Ke}y^{T}\right)}\;\in R^{1\times NK}$$

(5) $$\mathrm{Logits}=\alpha \times \mathrm{Similarity}\times \mathrm{Value}+\mathrm{Query}\times W^{T}$$

Furthermore, the Tip Clip model serves as a TCSMLP. The training of TCSMLP is governed by an objective function (Equation 6) featuring a dynamic regularization coefficient, *λ*(*t*) (Equation 7), which is adaptively adjusted based on feedback signals from the supervisor to improve model generalization:

(6) $$L=L_{\mathrm{MLP}}+\lambda \left(t\right)\times R\left(W\right)$$

(7) $$\lambda \left(t\right)=\lambda_{0}\;\left[1+\gamma \sum_{m=1}^{M}\beta_{m}F_{m}\left(t\right)\right]$$

This study also utilized ResNet [17, 18, 48], MLP [20], RFR [10, 49, 50], GBRT [19], SVR [51], XGBoost [52], CLIP [21, 32], TCSMLP, and Feature‐level fusion [29, 30, 31] to predict the grading of tea leaves across various dimensions. Detailed information on the model structure and model evaluation metrics can be found in the Supporting Information.

The proposed Long‐Tea‐CLIP framework adopts a modular architecture in which separate neural networks are designed to predict different sensory attributes of flat green tea. This design is primarily motivated by the traditional sensory evaluation procedure used by professional tea tasters, where tea quality is assessed across multiple dimensions (appearance, aroma, taste, soup color, and infused leaves). Each dimension reflects a relatively independent perceptual process while maintaining potential correlations with others. By assigning dedicated prediction modules to individual sensory attributes, our multimodal framework aims to mimic this expert evaluation process, thereby improving the interpretability and transparency of the AI‐based assessment system. Furthermore, the modular structure enables the model outputs to be directly associated with specific sensory dimensions, ensuring consistency with established tea evaluation standards.

Although this grading problem could theoretically be modeled using a unified end‐to‐end MTL framework with a shared backbone and task‐specific heads, such architectures may introduce task interference (e.g., negative transfer) due to the highly heterogeneous nature of sensory attributes. To circumvent this, separate neural networks were independently constructed and trained for each of the five sensory dimensions. This decoupled approach was specifically implemented to prevent cross‐modality interference between disparate inputs (e.g., visual imagery vs. mass spectrometry data) and to enforce task‐specific feature extraction. The independent modular outputs were subsequently integrated to systematically emulate the step‐by‐step analytical grading process of human experts.

All experiments were performed on a computer running Windows 10, with an 8‐core i7‐9750H CPU (2.6 GHz), 16 GB of DDR4 RAM, and an NVIDIA GeForce GTX 1060 GPU (6 GB video memory, CUDA 10.2.89). The PyTorch framework (version 1.10.2) based on Python 3.6.3 was used to deploy CNN models for stable performance. The libraries utilized for image preprocessing, feature extraction, and machine learning included py‐opencv 3.4.2, numpy 1.19.5, and scikit‐learn 0.24.2. The regression model converged after 200 training rounds for the aroma and taste dataset, while the classification model for the image dataset converged after ten training rounds. Parameters from the best‐performing model on the training set were saved for testing. Each training experiment was repeated five times with random initialization to minimize errors, and average results were reported.

### 4.7 Statistical Analysis

Raw data were first evaluated for outliers, and input features for the AI models were standardized (e.g., *Z*‐score normalization). Data from at least three biological replicates are expressed as mean ± standard deviation (SD). Specific sample sizes (*n*) are detailed in the respective figure legends. Statistical analyses were performed using GraphPad Prism 9 (GraphPad Software, USA) and SPSS 26 (IBM, USA). The Shapiro–Wilk and Levene's tests were used to verify data normality and homogeneity of variances, respectively. For normally distributed data, two‐group comparisons were conducted via two‐tailed Student's *t*‐tests, while multiple comparisons were analyzed using one‐way ANOVA followed by Tukey's posthoc test. All tests were two‐sided, with significance defined as *p < 0.05, **p < 0.01, ***p < 0.001, ****p < 0.0001, or n.s. (not significant).

For AI evaluation and multivariate analysis, machine learning performance (AUROC, RMSE, *R* ^2^) was validated using a fivefold cross‐validation procedure, which was repeated 20 times to ensure stability using Python 3.6.3. Metabolomics‐based multivariate analyses, including PCA and OPLS‐DA, were performed utilizing SIMCA 13.0 (Umetrics AB, Umea, Sweden) and MetaboAnalyst 6.0 (www.metaboanalyst.ca) with appropriate data scaling.

## Funding

Key Research and Development Program of Zhejiang Province 2025C01093 (Yanqun Xu). National Key Research and Development Program of China 2023YFD1601500 (Zhonghua Liu). The National Natural Science Foundation of China 32272773 (Zhonghua Liu). The Natural Science Foundation of Hunan Province 2023JJ40319 (Zhonghua Liu).

## Conflicts of Interest

The authors declare no known competing financial interests or personal relationships that could have appeared to influence the work reported in this paper.

## Supporting information




**Supporting File 1**: advs74970‐sup‐0001‐SuppMat.docx.

**Supporting File 2**: advs74970‐sup‐0002‐DataS1‐S6.zip.

**Supporting File 3**: advs74970‐sup‐0003‐SupplementaryTableS1‐S8.zip.

## Data Availability

The datasets generated and analyzed during the current study, as well as the source code for the Long‐Tea‐CLIP framework, are publicly available in the GitHub repository at https://github.com/2521614022/Long‐Tea. Further data are available within the main text and in the Supporting Information.

## References

[1] X. Zhang , “Tea and Cancer Prevention,” Journal of Cancer Research Updates 4, no. 2 (2015): 65.

[2] X. Zan , D. Yang , Y. Xiao , et al., “Facile General Injectable Gelatin/Metal/Tea Polyphenol Double Nanonetworks Remodel Wound Microenvironment and Accelerate Healing,” Advanced Science 11, no. 9 (2024): 2305405, 10.1002/advs.202305405. 38124471 PMC10916639

[3] S. Chen , P. Wang , W. Kong , et al., “Gene Mining and Genomics‐Assisted Breeding Empowered by the Pangenome of Tea Plant *Camellia sinensis* ,” Nature Plants 9, no. 12 (1986): 1986–1999, 10.1038/s41477-023-01565-z. 38012346

[4] Q. Xu , Y. Yang , K. Hu , et al., “Economic, Environmental, and Emergy Analysis of China's Green Tea Production,” Sustainable Production and Consumption 28 (2021): 269–280, 10.1016/j.spc.2021.04.019.

[5] M. A. Wouters , S. Iismaa , S. W. Fan , and N. L. Haworth , “Thiol‐Based Redox Signalling: Rust Never Sleeps,” International Journal of Biochemistry & Cell Biology 43, no. 8 (2011): 1079–1085, 10.1016/j.biocel.2011.04.002. 21513814

[6] A. Bhargava , A. Bansal , V. Goyal , and P. Bansal , “A Review on Tea Quality and Safety Using Emerging Parameters,” Journal of Food Measurement and Characterization 16, no. 2 (2022): 1291–1311, 10.1007/s11694-021-01232-x.

[7] J. C. Zhu , Y. Zhu , K. Wang , Y. W. Niu , and Z. B. Xiao , “Characterization of Key Aroma Compounds and Enantiomer Distribution in Longjing Tea,” Food Chemistry 361 (2021): 130096, 10.1016/j.foodchem.2021.130096. 34023691

[8] M. Li , T. Pan , and Q. Chen , “Estimation of Tea Quality Grade Using Statistical Identification of Key Variables,” Food Control 119 (2021): 107485, 10.1016/j.foodcont.2020.107485.

[9] M. Mauger , To Rub the Nose in the Tea″: Smell, Taste, and the Assessment of Quality in Early Nineteenth‐Century Tea Retail, ed. S. Dyer , (Springer International Publishing, 2022), 17–34.

[10] B. Wang , Y. Li , M. Zhou , et al., “Smartphone‐Based Platforms Implementing Microfluidic Detection With Image‐Based Artificial Intelligence,” Nature Communications 14, no. 1 (2023): 1341. 10.1038/s41467-023-36017-x PMC10007670 36906581

[11] X. Li , Y. Hu , X. Zhang , X. Shi , W. J. Parak , and A. Pich , “Transvascular Transport of Nanocarriers for Tumor Delivery,” Nature Communications 15, no. 1 (2024): 8172, 10.1038/s41467-024-52416-0. PMC11408679 39289401

[12] I. M. Nasir , A. Bibi , J. H. Shah , et al., “Deep Learning‐Based Classification of Fruit Diseases: An Application for Precision Agriculture,” Computers, Materials & Continua 66, no. 2 (2021): 1949–1962, 10.32604/cmc.2020.012945.

[13] F. Gao , J. Ding , B. Gai , et al., “Interpretable Multimodal Fusion Model for Bridged Histology and Genomics Survival Prediction in Pan‐Cancer,” Advanced Science 12, no. 17 (2025): 2407060, 10.1002/advs.202407060. 40051298 PMC12061278

[14] Z. W. Dou , M. X. Ji , M. Wang , and Y. N. Shao , “Price Prediction of Pu′er Tea Based on ARIMA and BP Models,” Neural Computing and Applications 34, no. 5 (2022): 3495–3511, 10.1007/s00521-021-05827-9. 33746365 PMC7960402

[15] Y. Ektefaie , G. Dasoulas , A. Noori , M. Farhat , and M. Zitnik , “Multimodal Learning With Graphs,” Nature Machine Intelligence 5, no. 4 (2023): 340–350, 10.1038/s42256-023-00624-6. PMC10704992 38076673

[16] Q. Q. Li , C. Y. Zhang , H. W. Wang , et al., “Machine Learning Technique Combined With Data Fusion Strategies: A Tea Grade Discrimination Platform,” Industrial Crops and Products 203 (2023): 117127, 10.1016/j.indcrop.2023.117127.

[17] M. Yu , W. Li , Y. Yu , et al., “Deep Learning Large‐Scale Drug Discovery and Repurposing,” Nature Computational Science 4, no. 8 (2024): 600–614, 10.1038/s43588-024-00679-4. 39169261

[18] K. He , X. Zhang , S. Ren , and J. Sun , “Deep Residual Learning for Image Recognition,” in Proceedings of the IEEE Conference on Computer Vision and Pattern Recognition (CVPR) (IEEE, 2016), 770–778, 10.1109/CVPR.2016.90.

[19] J. H. Friedman , “Greedy Function Approximation: A Gradient Boosting Machine,” Annals of Statistics 29, no. 5 (2001): 1189, 10.1214/aos/1013203451.

[20] A. Zhang , Z. C. Lipton , M. Li , and A. J. Smola , “Dive into Deep Learning,” *arXiv preprint:2106.11342*, 2021, 10.48550/arXiv.2106.11342.

[21] A. Radford , J. W. Kim , and C. Hallacy , “Learning Transferable Visual Models From Natural Language Supervision,” in International Conference on Machine Learning (PMLR, 2021), 8748.

[22] C. Zhang , J. Wang , G. Lu , S. Fei , T. Zheng , and B. Huang , “Automated Tea Quality Identification Based on Deep Convolutional Neural Networks and Transfer Learning,” Journal of Food Process Engineering 46, no. 4 (2023): 14303, 10.1111/jfpe.14303.

[23] X. Zhu , D. Shen , R. Wang , Y. Zheng , S. Su , and F. Chen , “Maturity Grading and Identification of Camellia Oleifera Fruit Based on Unsupervised Image Clustering,” Foods 11, no. 23 (2022): 3800, 10.3390/foods11233800. 36496609 PMC9736105

[24] C. Liu , W. Lu , B. Gao , H. Kimura , Y. Li , and J. Wang , “Rapid Identification of Chrysanthemum Teas by Computer Vision and Deep Learning,” Food Science & Nutrition 8, no. 4 (2020): 1968–1977, 10.1002/fsn3.1484. 32328263 PMC7174232

[25] Y. Peng , C. Zheng , S. Guo , et al., “Metabolomics Integrated With Machine Learning to Discriminate the Geographic Origin of Rougui Wuyi Rock Tea,” Npj Science of Food 7, no. 1 (2023): 7, 10.1038/s41538-023-00187-1. 36928372 PMC10020150

[26] C. Y. Peng , Y.‐F. Ren , Z.‐H. Ye , et al., “A Comparative UHPLC‐Q/TOF‐MS‐Based Metabolomics Approach Coupled With Machine Learning Algorithms to Differentiate Keemun Black Teas From Narrow‐Geographic Origins,” Food Research International 158 (2022): 111512, 10.1016/j.foodres.2022.111512. 35840220

[27] J. F. Tan , U. H. Engelhardt , Z. Lin , N. Kaiser , and B. Maiwald , “Flavonoids, Phenolic Acids, Alkaloids and Theanine in Different Types of Authentic Chinese White Tea Samples,” Journal of Food Composition and Analysis 57 (2017): 8–15, 10.1016/j.jfca.2016.12.011.

[28] Y. He , Q. F. Zhang , A. C. Inostroza , et al., “Application of Metabolic Fingerprinting in Tea Quality Evaluation,” Food Control 160 (2024): 110361, 10.1016/j.foodcont.2024.110361.

[29] Z. Shan , Y. Zhang , Q. Yang , et al., “Contrastive Pre‐Training With Multi‐View Fusion for No‐Reference Point Cloud Quality Assessment,” in Proceedings of the IEEE/CVF Conference on Computer Vision and Pattern Recognition (IEEE, 2024), 25942–25951.

[30] S. Mo and P. Morgado , “Unveiling the Power of Audio‐Visual Early Fusion Transformers With Dense Interactions Through Masked Modeling,” in Proceedings of the IEEE/CVF Conference on Computer Vision and Pattern Recognition (IEEE, 2024), 27186–27196.

[31] Z. Lv , Y. Wei , W. Zuo , and K.‐Y. K. Wong , “PLACE: Adaptive Layout‐Semantic Fusion for Semantic Image Synthesis,” in Proceedings of the IEEE/CVF Conference on Computer Vision and Pattern Recognition (IEEE, 2024), 9264–9274.

[32] J. Duan , L. Lai , Z. Yang , Z. Luo , and H. Yuan , “Multi‐Feature Language‐Image Model for Fruit Quality Image Classification,” Computers and Electronics in Agriculture 227 (2024): 109462, 10.1016/j.compag.2024.109462.

[33] L. Q. Li , Y. R. Chen , S. Dong , et al., “Rapid and Comprehensive Grade Evaluation of Keemun Black Tea Using Efficient Multidimensional Data Fusion,” Food Chemistry: X 20 (2023): 100924, 10.1016/j.fochx.2023.100924. 38144790 PMC10740040

[34] M. Jang , G. Bae , Y. M. Kwon , et al., “Artificial Q‐Grader: Machine Learning‐Enabled Intelligent Olfactory and Gustatory Sensing System,” Advanced Science 11, no. 23 (2024): 2308976, 10.1002/advs.202308976. 38582529 PMC11186046

[35] G. X. Ren , R. Wu , L. L. Yin , Z. Z. Zhang , and J. M. Ning , “Description of Tea Quality Using Deep Learning and Multi‐Sensor Feature Fusion,” Journal of Food Composition and Analysis 126 (2024): 105924, 10.1016/j.jfca.2023.105924.

[36] X. Y. Zhao , Y. He , H. Zhang , Z. Ding , C. Zhou , and K. Zhang , “A Quality Grade Classification Method for Fresh Tea Leaves Based on an Improved YOLOv8x‐SPPCSPC‐CBAM Model,” Scientific Reports 14, no. 1 (2024): 4166, 10.1038/s41598-024-54389-y. 38378791 PMC10879108

[37] H. J. Wang , J. A. Gu , and M. N. Wang , “A Review on the Application of Computer Vision and Machine Learning in the Tea industry,” Frontiers in Sustainable Food Systems 7 (2023): 1172543, 10.3389/fsufs.2023.1172543.

[38] H. R. Tan , L. Y. Chan , A. Ong , Y.‐Q. Xu , X.‐B. Zhang , and W. Zhou , “Atmospheric Solids Analysis Probe‐Mass Spectrometry (ASAP‐MS) as a Rapid Fingerprinting Technique to Differentiate the Harvest Seasons of Tieguanyin Oolong Teas,” Food Chemistry 408 (2023): 135135, 10.1016/j.foodchem.2022.135135. 36527922

[39] C. Wang , J. Yang , and Q. Wu , “A Global Extended Extreme Learning Machine Combined With Electronic Nose for Identifying Tea Gas Information,” Measurement and Control 55, no. 7–8 (2022): 746–756, 10.1177/00202940221090973.

[40] R. C. Zhi , L. Zhao , and D. Z. Zhang , “A Framework for the Multi‐Level Fusion of Electronic Nose and Electronic Tongue for Tea Quality Assessment,” Sensors 17, no. 5 (2017): 1007, 10.3390/s17051007. 28467364 PMC5469530

[41] J. K. Carrillo , C. M. Durán , J. M. Cáceres , et al., “Assessment of E‐Senses Performance Through Machine Learning Models for Colombian Herbal Teas Classification,” Chemosensors 11, no. 7 (2023): 354, 10.3390/chemosensors11070354.

[42] S. F. Zhang , D. H. Zhu , and X. J. Chen , “Analysis of E‐Tongue Data for Tea Classification Based on Semi‐Supervised Learning of Generative Adversarial Network,” Chinese Journal of Analytical Chemistry 50, no. 2 (2022): 77–85, 10.1016/j.cjac.2021.11.008.

[43] S. Y. Gong , Y. Zhao , and C. Lu , GBT23776‐2018 Methodology for Sensory Valuation of Tea (China Standards Press, 2018).

[44] Y. Zhao , T. Shen , H. Huang , et al., TXHLJ 001‐2021 Hangzhou West Lake Longjing Tea Management Association Group Standard, (National Public Service Platform for Standards Information, Hangzhou, China, 2021).

[45] S. C. Wong , A. Gatt , V. Stamatescu , and M. D. McDonnell , “Understanding Data Augmentation for Classification: When to Warp,” in 2016 International Conference on Digital Image Computing: Techniques and Applications (DICTA, IEEE, 2016), 1–6.

[46] J.‐Q. Wang , Y.‐Q. Fu , J.‐X. Chen , et al., “Effects of Baking Treatment on the Sensory Quality and Physicochemical Properties of Green Tea With Different Processing Methods,” Food Chemistry 380 (2022): 132217, 10.1016/j.foodchem.2022.132217. 35101788

[47] C. Shao , Z. Deng , J. Liu , et al., “Effects of Preharvest Shading on Dynamic Changes in Metabolites, Gene Expression, and Enzyme Activity of Three Tea Types During Processing,” Journal of Agricultural and Food Chemistry 70, no. 45 (2022): 14544–14558, 10.1021/acs.jafc.2c05456. 36321848

[48] X. C. Zhang and Y. Wang , “AI‐Recognized Mitochondrial Phenotype Enables Identification of Drug Targets,” Nature Computational Science 4, no. 8 (2024): 563–564, 10.1038/s43588-024-00682-9. 39174760

[49] C. I. Allen Akselrud , “Random Forest Regression Models in Ecology: Accounting for Messy Biological Data and Producing Predictions With Uncertainty,” Fisheries Research 280 (2024): 107161, 10.1016/j.fishres.2024.107161.

[50] L. Liu , M. Bi , Y. Wang , et al., “Artificial Intelligence‐Powered Microfluidics for Nanomedicine and Materials Synthesis,” Nanoscale 13, no. 46 (2021): 19352–19366, 10.1039/D1NR06195J. 34812823

[51] A. J. Smola and B. Schölkopf , “A Tutorial on Support Vector Regression,” Statistics and Computing 14, no. 3 (2004): 199–222, 10.1023/B:Stco.0000035301.49549.88.

[52] T. Q. Chen and C. Guestrin , “XGBoost: A Scalable Tree Boosting System,” in Proceedings of the 22nd Acm Sigkdd International Conference on Knowledge Discovery and Data Mining (Association for Computing Machinery, 2016), 785–794.

