# Supplementary material for: Long‐Tea‐CLIP: An Expert‐Level Multimodal AI Framework for Fine‐Grained Green Tea Grading Across Five Sensory Dimensions
Source: Adv Sci (Weinh). 2026 Mar 27;13(32):e18235. doi: 10.1002/advs.202518235 (PMC13252660; doi:10.1002/advs.202518235)
Supplement: Supplementary file 1 — Supporting File 1: advs74970‐sup‐0001‐SuppMat.docx. [file ADVS-13-e18235-s001.docx]

Supplementary Materials for

**Long-Tea-CLIP: A Multimodal AI Framework Replacing Tea Masters in Grading Flat-Shaped Green Tea Based on Five Sensory Dimensions**

Yanqun Xu ^#^, Zhengfang Xue ^#^, Qing Luo, Xu Liu, Yongquan Xu, Huang Jing, Xingcai Zhang, Xinyuan Zhang *, Zhonghua Liu * & Zisheng Luo *

*Corresponding author. Email: luozisheng@zju.edu.cn

**This PDF file includes:**

Supplementary Text: Methods

Figs. S1 to S7

**Other Supplementary Materials for this manuscript include the following:**

Data S1 to S6

Tables S1 to S8

Supplementary Text

**1. Method**

**1.1 AI Models**

*1.1.1 ResNet*

ResNet (Residual Network) is a deep neural network architecture introduced by Kaiming He et al. in 2015 at Microsoft Research Asia. ResNet addresses the issues of vanishing and exploding gradients during deep network training by introducing residual connections, which effectively increase the network's depth and improve its performance. The core concept of ResNet is the addition of shortcut connections, allowing input information to bypass intermediate layers and be passed directly to subsequent layers. This mechanism ensures that if deeper layers fail to learn useful features, the model can still rely on the information from earlier layers, preventing network performance degradation. Residual connections enable the network to focus on learning only the residuals (i.e., the difference between the input and output), simplifying the learning process，the formula is as follows：

$$h^{l+1}=h^{l}+\sigma\left( W^{l}h^{l}+b^{l} \right)$$

Where $h^{l}$ is the input of the l-th layer, $h^{l+1}$ is the output of the l-th layer, $W^{l}$ is the weight of the l-th layer convolution kernel, $\sigma$ is the activation function of the l-th layer, and $b^{l}$ is the bias of the l-th layer convolution kernel.

The cost function $\mathcal{L}$ of the ResNet model typically uses the **cross-entropy loss**, defined as:

$$\mathcal{L=-}\frac{1}{N}\sum_{i=1}^{N} \sum_{c=1}^{C} y_{c}^{i}\log p_{c}^{i}$$

Where, N is the total number of samples involved in training, C is the number of classes, $y_{c}^{i}$ indicating whether the ith sample belongs to class C (if so, the value is 1, otherwise, it is 0), and $p_{c}^{i}$ indicating the probability that the ith sample belongs to class C in the model output.

ResNet consists of multiple residual blocks connected in series. Each block has two paths: the identity mapping, which passes input information directly to the output, and the residual mapping, which transforms the input before combining it with the identity mapping's output. This structure facilitates gradient flow during backpropagation, mitigating the issue of vanishing or exploding gradients.

In this study, ResNet and its extended models were selected for classifying tea shape data to verify the effectiveness of the residual structure. The architecture used was ResNet-18, which includes an input layer (224 × 224 × 3), a convolutional layer (7 × 7, 64 filters, stride 2, output size 112 × 112), a max-pooling layer (3 × 3, stride 2, output size 56 × 56), and convolutional layers with increasing filter sizes (64, 128, 256, 512 filters) that progressively reduce the output size. An average pooling layer and fully connected layer follow, with an output size of 1×3 for classification. The model's floating-point operations were approximately 1.8×10^9^.

The ResNet-18 model was pre-trained on the ImageNet-1K dataset. After loading the pre-trained parameters, the model was fine-tuned by the stacked tea images dataset, which contains 5,822 training images and 1,941 test images. Training hyperparameters were set as follows: learning rate = 5e^-5^, batch size = 128, and 10 training epochs. During the training process, a strategy of dynamically adjusting the learning rate is adopted. Set the learning rate decay parameter, and decay the learning rate when the number of training rounds reaches the preset threshold, so that the model can effectively tend to converge during the training process. Data augmentation techniques, such as 90° rotations, vertical and horizontal flips, and 180° rotations, were applied to increase the training set size fivefold. These measures ensured robust model training and improved classification accuracy and generalization ability.

After training each classification model for evaluating tea appearance, the parameters associated with the highest classification accuracy on the validation set were saved. These models were subsequently used to perform inference on individual tea appearance images, generating an evaluation descriptor sequence for each image. This process was applied to the entire dataset of tea appearance images, resulting in multiple descriptor sequences that formed a matrix. To establish a conversion between tea appearance descriptors and appearance scores, a pre-constructed database was employed. This database contained descriptors and their respective scores across various categories, facilitating the conversion of the descriptor matrix into a score sequence. Specifically, seven evaluation dimensions were used for piled tea appearance: straightness, smoothness, tenderness, greenness, luster, color uniformity, and integrity. Each dimension included several categories, totaling 46 categories. Thus, each processed piled tea image produced a 1×46 descriptor sequence vector. The complete piled tea appearance dataset contained 7,763 images, resulting in a final 7,763×46 matrix. Using the descriptor-score database, matrix operations converted this matrix into a 7,763×1 score sequence, with each entry representing the appearance score for a corresponding image.

*1.1.2. Multilayer Perceptron (MLP)*

A multilayer perceptron (MLP) is a fundamental artificial neural network (ANN) architecture consisting of multiple layers: an input layer, one or more hidden layers, and an output layer. MLP is a feed-forward network, meaning information flows unidirectionally from input to output, with all layers fully connected. The combination of multilayer neurons and nonlinear activation functions enables the MLP to learn complex relationships between inputs and outputs.

The learning process occurs through forward propagation and backpropagation. During forward propagation, the input signal passes through the hidden layers to produce the model's prediction. Backpropagation calculates the gradient of the loss function with respect to the model parameters, allowing for weight updates. Through iterative forward and backpropagation, the model adjusts its weights and biases, refining predictions to match true values，the formula is as follows：

$$h^{l+1}=\sigma\left( W^{l}h^{l}+b^{l} \right)$$

Where $h^{l}$ is the input of the l-th layer, $h^{l+1}$ is the output of the l-th layer, $W^{l}$ is the weight of the l-th layer convolution kernel, $\sigma$ is the activation function of the l-th layer, and $b^{l}$ is the bias of the l-th layer convolution kernel.

The objective function of the MLP is designed based on the **Mean Squared Error (MSE)**, which measures the average squared difference between predicted and true values. The loss function is defined as:

$$\mathcal{L=}\frac{1}{2N}\sum_{i=1}^{N} \left( y_{i}-\hat{y}_{i} \right)^{2}$$

Where N is total number of samples，$y_{i}$ is the ground truth (target) value of the i-th sample，$\hat{y}_{i}$ is the predicted value for the i-th sample，$\left( y_{i}-\hat{y}_{i} \right)^{2}$ is squared prediction error for sample i.

In this study, a custom MLP structure was selected as a regression model for GC-MS and metabolomics data to assess its effectiveness. For metabolomics data, the architecture included an input layer (18 features), three hidden layers (256, 128, 64 nodes), and an output layer. The GC-MS model had an input layer (312 features), three hidden layers (209, 140, 94 nodes), and an output layer. For appearance of dispersed tea, the architecture included an input layer (20 features), three hidden layers (256, 128, 64 nodes), and an output layer. Training parameters for both models were set to num_epochs = 200, learning_rate = 0.01 (metabolomics) or 0.001 (GC-MS), weight_decay = 0.003, and batch_size = 16. Five-fold cross-validation was applied to ensure robustness.

*1.1.3. Random Forest Regression*

Random Forest Regression is an ensemble learning algorithm that improves performance and robustness by constructing multiple decision trees and aggregating their predictions. Each decision tree in the forest is independent, and the final prediction is based on the collective output of all trees. In this study, Random Forest Regression was chosen as one of the regression models for GC-MS and metabolomics data to evaluate the method's effectiveness. The formula is as follows:

$$\hat{\text{y}}\text{=}\frac{\text{1}}{\text{T}}\sum_{\text{t=1}}^{\text{T}} \hat{y}_{t}\text{(X)}$$

Where $\hat{y}_{t}$ is the prediction result of the t-th decision tree, T is the number of decision trees, and $\hat{\text{y}}$ is the final prediction result of the model for the input sample X. By combining multiple decision trees, random forest significantly improves the prediction accuracy of the model, and at the same time, the model also has the ability to deal with higher dimensional feature data. Because each decision tree splits the regression tree using a set of randomly extracted features, the introduced randomness enhances the resistance to overfitting, and thus enables better generalization to unknown data. However, because the random forest needs to train multiple decision trees, the computational cost is high, which is particularly evident when the amount of data is large.

Key parameters for optimizing Random Forest Regression include:

n_estimators: Number of decision trees (more trees generally improve performance but increase complexity).

max_depth: Maximum depth of the trees, which prevents overfitting.

min_samples_leaf: Minimum number of samples required at a leaf node to avoid overfitting.

max_features: Number of features considered when splitting a node, controlling randomness in feature selection.

In this experiment, the parameters were set as follows: n_estimators = 100, max_depth = 5, min_samples_leaf = 1, max_features = 1.

*1.1.4.* *Gradient Boosting Regression Tree (GBRT)*

GBRT is an ensemble learning algorithm that iteratively builds decision trees and combines their predictions to create a stronger regression model. Each subsequent tree is trained on the residuals (the difference between actual and predicted values) of previous trees. The final prediction is a weighted average of all trees' predictions.

$$\hat{y}_{i}^{m}=\hat{y}_{i}^{m-1}+\eta h_{m}(X_{i})$$

Where, $\hat{y}_{i}^{m}$ is the prediction value of the current iteration of the regression tree, $\hat{y}_{i}^{m-1}$ is the prediction value of the previous iteration, $h_{m}(X_{i})$ is the new regression tree generated by fitting the prediction residual $r_{i}^{m}$ of the previous iteration, and $\eta$ is the influence weight of the new regression tree. The negative gradient, or pseudo-residual, is calculated for each sample *i* as:

$$r_{i}^{m}=-\left[ \frac{\partial L(y_{i},\hat{y}_{i}^{m-1})}{\partial\hat{y}_{i}^{m-1}} \right]$$

Key parameters for optimizing GBRT include:

n_estimators: Number of trees (increasing trees enhances performance but increases complexity).

learning_rate: Controls the contribution of each tree to the final prediction.

max_depth: Limits the depth of each tree to prevent overfitting.

In this experiment, the parameters were set as follows: n_estimators = 100, learning_rate = 0.05, max_depth = 3.

*1.1.5. Support Vector Regression (SVR)*

SVR, based on the Support Vector Machine (SVM), is a powerful regression model with strong generalization capabilities, especially for small sample sizes and high-dimensional data. SVR finds the optimal hyperplane in a high-dimensional feature space to predict continuous values, maximizing the margin between the hyperplane and sample data.

Key parameters for fine-tuning SVR include:

Kernel: Maps input data to a higher-dimensional space for non-linear separability.

C (Penalty Parameter): Controls the trade-off between model complexity and training error.

epsilon: Defines the error tolerance.

gamma: For RBF kernels, controls the influence range of a single training sample.

In this experiment, the parameters were: kernel = 'rbf', C = 100, gamma = 0.5, epsilon = 0.2. The SVR model minimizes the regularized empirical risk using the ε-insensitive loss function, subject to the constraints on prediction deviations. The prediction function takes the form:

$$\hat{y}=\sum_{i=1}^{n} (a_{i}-a_{i}^{*})K(X_{i},X)+b$$

Where $a_{i}$ and $a_{i}^{*}$ are the Lagrangian operators derived from the support vectors, $K(X_{i},X)$ is the kernel function, and $b$ is the bias term.

*1.1.6. XGBoost*

XGBoost (eXtreme Gradient Boosting) is a high-performance gradient boosting framework that iteratively builds decision trees to enhance prediction accuracy. Each tree attempts to correct the errors of the previous ones. XGBoost incorporates optimizations such as regularization to prevent overfitting.

$$\mathcal{L}=\sum_{i=1}^{n} loss(y_{i},\hat{y}_{i})+\sum_{k=1}^{K} \Omega(f_{k})$$

Where, $loss$ is the loss function,，$\Omega(f_{k})$ is the regularization term，*f_k_* represents the *k*-th decision tree，K is the number of trees，n is the number of training samples，$y_{i}$ is the true label of the *i*-th sample，$\hat{y}_{i}$is the model prediction for the *i*-th sample.

To control the model complexity and prevent overfitting, the regularization term is defined as:

$$\Omega(f_{k})=\gamma T+\frac{1}{2}\lambda\parallel\omega\parallel^{2}$$

where，T is the number of leaf nodes in the tree，γ is the penalty coefficient for the number of leaf nodes，ω denotes the vector of leaf weights，λ is the regularization coefficient for the leaf weights.

Key parameters for XGBoost include:

max_depth: Maximum tree depth (increasing depth increases complexity and risk of overfitting).

eta (learning_rate): Controls the step size for weight updates.

num_round: Number of boosting rounds.

In this experiment, the parameters were set to: max_depth = 3, eta = 0.1, num_round = 100, and the loss function was reg

Here is the polished version of the paragraph with improvements in academic style, grammar, clarity, conciseness, and overall readability.

*1.1.7. Feature-level fusion*

We perform feature-level fusion on sequence and image multimodal data. The image data is extracted as image features by the image encoder (residual convolutional network), and the sequence data is extracted as sequence features by the sequence encoder (MLP). After the fusion of the two, they are taken as K and V, and cross attention is performed with the fusion feature Q of the image sequence. Then, the tea quality score is output through the FC layer.

*1.1.8. Tip Clip*

The CLIP (Contrastive Language-Image Pre-Training) model is a multimodal pre-trained neural network released by OpenAI in 2021. It represents an effective and easily transferable method for learning from natural language supervision. The foundational CLIP model undergoes pre-training using a vast scale of 400 million text-image pairs. The core idea of the model is to learn the alignment between images and text using a large amount of paired data of images and text. The model comprises two main components: a Text Encoder, which converts text into low-dimensional vector representations, and an Image Encoder, which converts images into similar vector representations. During the prediction phase, the CLIP model generates predictions by calculating the cosine similarity between text and image vectors, making CLIP particularly suitable for zero-shot and few-shot learning tasks.

For the zero-shot classification of a tea quality grading dataset (including appearance images, GCMS data, metabolomics data, liquor color images, and leaf base images), the latter four types of data can be converted into feature vectors of specific dimensions through feature engineering and coupled with the output of the appearance images from the Image Encoder to obtain a new encoded vector I_1_. The text encoding vectors T_1_, T_2_, and T_3_ for special-grade tea, first-grade tea, and second-grade tea are output through the Text Encoder. The cosine similarity between I_1_ and T_1_, T_2_, T_3_ is calculated, and the category corresponding to the highest text encoding vector is taken as the category of the current combined input data.

The pain point lies in that the foundational CLIP model, pre-trained on 400 million text-image pairs, may not have learned specialized data such as tea quality grades, resulting in low classification accuracy. To further enhance CLIP's transfer performance while considering both time and space resource costs, this paper introduces a few-shot image classification method that improves CLIP's accuracy in downstream tasks without requiring additional downstream training, which is called Tip Clip. This method leverages CLIP to construct a Cache Model in a non-training manner to store classification knowledge from downstream training data, here referring to tea quality grading knowledge. Subsequently, during testing, more reliable classification results are obtained by linearly summing the predictions of the Cache Model and the foundational CLIP model.

The working process of the multi-modal Longjing tea classification model comprises the following steps of:

Constructing a multi-modal ensemble learning supervisor, comprising: building a buffer based on the collected small sample image-text data set, embedding and coding the image data of each category by using a Visual Encoder module of the pre-training CLIP, and constructing a buffer index as shown in the formula:

$$Key=VisualEncoder(I_{k})\in R^{NK\times C}$$

Where Key represents the key vector in the image buffer, VisualEncoder is the visual encoder module in the CLIP model, which is used to extract the features of the image sample, R represents the real number field, indicating that the value of Key is in the real number range, $I_{k}$ is the image input of the kth category or sample, N represents the number of categories, The number of rows represents the total number of classes multi the number of image samples in each class, and the number of samples in total; C represents the embedded feature dimension output by the vision encoder, that is, the vector dimension of each image after being encoded.

OneHot is used to encode the text label corresponding to each category of image, and the key value of the buffer is constructed as shown in the following formula:

$$Value=OneHot(L_{k})\in R^{NK\times N}$$

Where, Value represents the value vector in the image buffer, OneHot represents the one-hot coding module for one-hot coding the class labels, $L_{k}$ is the text label or class label corresponding to the kth image sample, N represents the number of classes, K represents the number of images of each class participating in the buffer construction, and the coding result is a matrix of dimension NK × N.

$$Query=VisualEncoder(I)\in R^{1\times C}$$

Where, Query represents the query vector obtained after the image to be recognized is extracted by the vision encoder, and I represents the input image code; 1 represents that there is only one input image sample at present, and C represents the embedded feature dimension output by the vision encoder, so the final output is an embedded vector with a dimension of 1 × C.

As for an image to be predict, inputting that image to be predict into a visual encoder to obtain a corresponding embedded coding vector as an inquiry value. calculate the similarity between a buffer index and the inquiry value by using the following formula, and taking a key value from the buffer for decoding on the basis of the similarity:

$$Similarity=e^{-\beta(1-Query\times{Key}^{T})}\in R^{1\times NK}$$

Where, Similarity represents the similarity between the query vector and the key vector, $\beta$ is the scaling coefficient, which is used to adjust the variation range of the similarity function, ${Key}^{T}$ represents the transpose of the Key. $Query$ × ${Key}^{T}$ is the dot product similarity. Therefore, the dimension of the final output Similarity is 1 × N × K.

Based on the result of decoding, the classifier in the weighted combined pre-training CLIP model performs zero-sample prediction on the image data as the label corresponding to the ensemble learning supervisor:

$$Logits=\alpha\times Similarity\times Value+Query\times W^{T}$$

Where, $Logits$ represents the final prediction output vector, which is used to judge the corresponding label in supervised learning. $\alpha$ is the regularization coefficient, which is used to balance the contribution of the similarity-weighted label and the CLIP original classifier. $W^{T}$ is the weight transpose matrix of the CLIP model classifier, which is used to map Query to the prediction score of each class.

Let the total objective function of the TCSMLP model be $\mathcal{L}=\mathcal{L}_{\mathrm{MLP}}+\lambda(t)\mathcal{\times R(}W)$, where $\mathcal{L}_{\mathrm{MLP}}$ is the task loss, that is, the mean square error used in the MLP regression, $\mathcal{R(}W)$ is the regularization term, and $\lambda(t)$ is the dynamic adjustment coefficient, which is controlled by the feedback signal of the supervisor; define the construction method of the dynamic coefficient as follows:

$$\lambda(t)=\lambda_{0}\left[ 1+\gamma\sum_{m=1}^{M} \beta_{m}F_{m}(t) \right]$$

Where, $\lambda_{0}$ denotes the initial regularization coefficient, $\gamma$ denotes the feedback sensitive coefficient, M denotes the number of regularization coefficient influence factors, $\beta_{m}$ denotes the weight of the mth influence factor, and $F_{m}(t)$ denotes the feedback function of the mth influence factor at time step t.

When m = 1, the feedback function $F_{m}(t)$ is defined as follows:

$$F_{m}(t)=\frac{\left| \mathcal{L}_{m}(t)-\mathcal{L}_{m}(t-1) \right|}{\mathcal{L}_{m}(t-1)}sign(\mathcal{L}_{m}(t)-\mathcal{L}_{m}(t-1))$$

For m = 2, the feedback function $F_{m}(t)$ is defined as follows:

$$F_{m}(t)=\frac{\left| \mathcal{L}_{m}(t)-\mathcal{L}_{m}(t-1) \right|}{\mathcal{L}_{m}(t-1)}sign'({Label}_{supervisor}-{Label}_{pred})$$

Where, $\mathcal{L}_{m}(t)$ represents the loss function value of TCSMLP at time step t, $sign$ is the standard signal function, ${Label}_{supervisor}$ represents the label recorded by the supervisor, ${Label}_{pred}$ represents the label of the current output of the model, and $sign'$ is the signal function based on the Boolean value. When $\mathcal{L}_{m}(t)$ is increased or the output of TCSMLP is inconsistent with the label recorded by the supervisor, it takes a positive value to enhance the regularization, otherwise, it takes a negative value to reduce the regularization.

**1.2. Performance Evaluation**

*1.2.1. Precision, Recall, F1-score, and Accuracy*

Precision, in machine learning evaluation, measures the proportion of correctly predicted positive samples out of all samples predicted as positive. Recall, also known as the sensitivity or true positive rate, measures how many true positive samples were correctly identified by the model. The F1-score is a comprehensive metric that balances precision and recall, calculated as their harmonic mean. The F1-score ranges from 0 to 1, with 1 indicating perfect model performance. Accuracy measures the overall effectiveness of a classification model, representing the ratio of correctly predicted samples to the total number of samples. The formulas for these metrics are as follows:

$$precision=\frac{\mathrm{TP}}{TP+FP}$$

$$recall=\frac{\mathrm{TP}}{TP+FN}$$

$${F1}_{score}=\frac{2\times precison\times recall}{precision+recall}$$

$$accuracy=\frac{TP+TN}{TP+FP+TN+FN}$$

Where:

- TP (True Positive): Correctly predicted positive samples.

- FP (False Positive): Incorrectly predicted positive samples.

- TN (True Negative): Correctly predicted negative samples.

- FN (False Negative): Incorrectly predicted negative samples.

*1.2.2. Macro Average*

The macro average (macro avg) is used to evaluate performance in multi-class classification problems by computing the average of metrics (e.g., precision, recall, F1-score) for each class independently. It does not consider the sample size for each class, treating all classes equally, which is useful when dealing with class imbalances.

$${precision}_{avg}=\frac{1}{N}\sum_{i=1}^{N} {precision}_{i}$$

$${recall}_{avg}=\frac{1}{N}\sum_{i=1}^{N} {recall}_{i}$$

Where:

${precision}_{i}$ and ${recall}_{i}$ represent the precision and recall of the i-th class, respectively, and N is the total number of classes. A confusion matrix can visually represent classification results, with each row showing the true class and each column showing the predicted class.

*1.2.3. Weighted Average*

The weighted average (weighted avg) accounts for the sample size in each class when calculating performance metrics (e.g., precision, recall, F1-score). It provides an overall average by weighting each class’s performance based on its proportion in the total sample.

$$\mathrm{weight}_{\mathrm{avg}}\text{=}\frac{\sum_{i=1}^{N} (\mathrm{value}_{i}\times\mathrm{weight}_{i})}{\sum_{i=1}^{N} \mathrm{weight}_{i}}$$

Where, $\mathrm{value}_{i}$ is the performance metric for the i-th class, and $\mathrm{weight}_{i}$ is the proportion of that class in the dataset.

*1.2.4. Cross Entropy Loss*

Cross entropy loss is a widely used loss function for classification tasks. It measures the divergence between the true probability distribution and the predicted distribution. For binary classification, the cross entropy loss for a single sample is calculated as:

$$loss=-[ylog(\hat{y})+(1-y)log(1-\hat{y})]$$

Where, $\mathrm{loss}$ is the loss fuction, y is the true label (0 or 1), and $\hat{y}$ is the predicted probability. For multi-class classification, the formula becomes:

$$\mathcal{L}_{\mathrm{CE}}=-\frac{1}{N}\sum_{i=1}^{N} [y_{i}log(\hat{y}_{i})+(1-y_{i})log(1-\hat{y}_{i})]$$

Where, N is the number of samples, y_i_ indicates whether the i-th sample, and $\hat{y}_{i}$ is the predicted probability.

*1.2.5. Logarithmic Root Mean Squared Error*

Logarithmic Root Mean Squared Error is a regression evaluation metric. In certain situations, especially when the target variable has a large range or a skewed distribution, using logarithmic error instead of raw error may be more appropriate for assessing model performance. Logarithmic error focuses more on relative error rather than absolute error, making the evaluation process more balanced. By reducing the influence of large target variable values, it emphasizes relative differences. This is particularly useful when the target variable spans a wide range or exhibits skewness. The square root operation amplifies small errors, prompting the model to pay more attention to samples with larger errors, thus improving its overall fit to the data.

$$\mathcal{L}_{\mathrm{RMSE}}=\sqrt{\frac{1}{N}\sum_{i=1}^{N} {(y_{i}-\hat{y}_{i})}^{2}}$$

Where, N is the number of samples, $y_{i}$ is the log-true value for the i-th sample, and $\hat{y}_{i}$ is the corresponding log-predicted value.

*1.2.6. R^2^*

Traditional regression typically uses the score function in scikit-learn to calculate the correlation between predicted and true values in regression models, represented by the R² score. The R² score can be interpreted as the proportion of variance in the dependent variable that is explainable by the model's predictions. It is calculated by dividing the squared error between predicted and true values by the total squared deviation of the true values. The R² score ranges from [0, 1], with values closer to 1 indicating better model performance. In the perceptron model, a custom implementation of R² score calculation is used. Formula for calculation:

$$\bar{y_{i}}=\frac{1}{N}\sum_{i=1}^{N} y_{i}$$

$$R_{score}^{2}=1-\frac{{\sum_{i=1}^{N} (\hat{y}_{i}-y_{i})}^{2}}{{\sum_{i=1}^{N} (y_{i}-\bar{y_{i}})}^{2}}$$

Fig. S1.


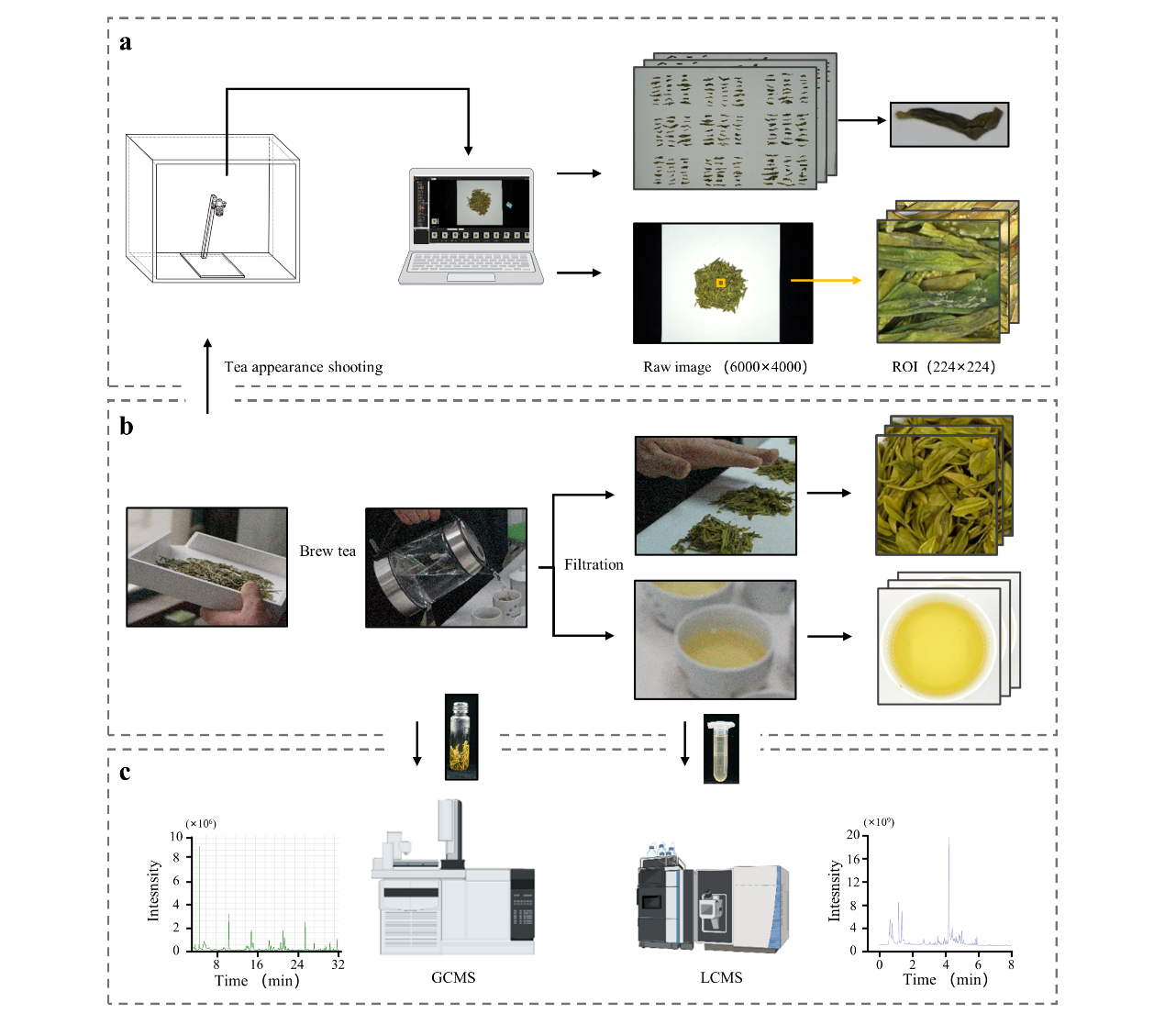


Supplementary Figure 1. Schematic illustration of the data collection workflow. (a) Imaging setup and region-of-interest (ROI) extraction for dispersed and stacked tea samples. (b) Preparation of brewed tea samples and image acquisition for infused leaves and soup color. (c) Acquisition workflow for aroma- and taste-related chemical data, including GC-MS analysis of volatile organic compounds (VOCs) from brewed tea and LC-MS-based metabolite profiling. Created in BioRender. Huang, J. (2026) https://BioRender.com/0dc34qe

Fig. S2.


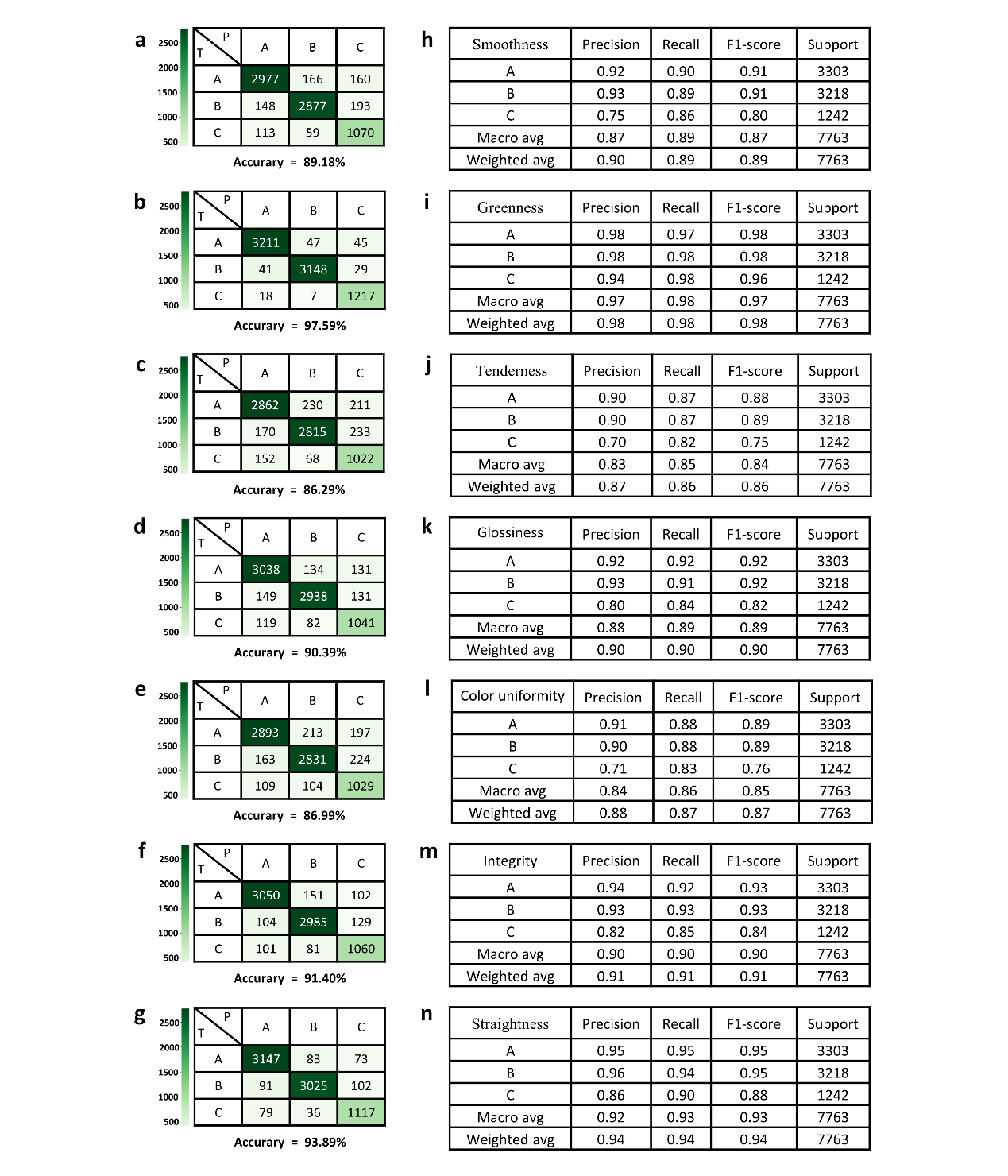


Supplementary Fig. 2. Confusion matrix of 7 sub-dimension of tea appearance:

(a) smoothness, (b) greenness, (c) tenderness, (d) glossiness, (e) uniformity of color, (f) Integrity, and (g) straightness respectively. (h–n) Corresponding classification metrics, including precision, recall, F1-score, and support, for each sub-dimension, evaluated on the same dataset (total n = 7,763)Fig. S3.


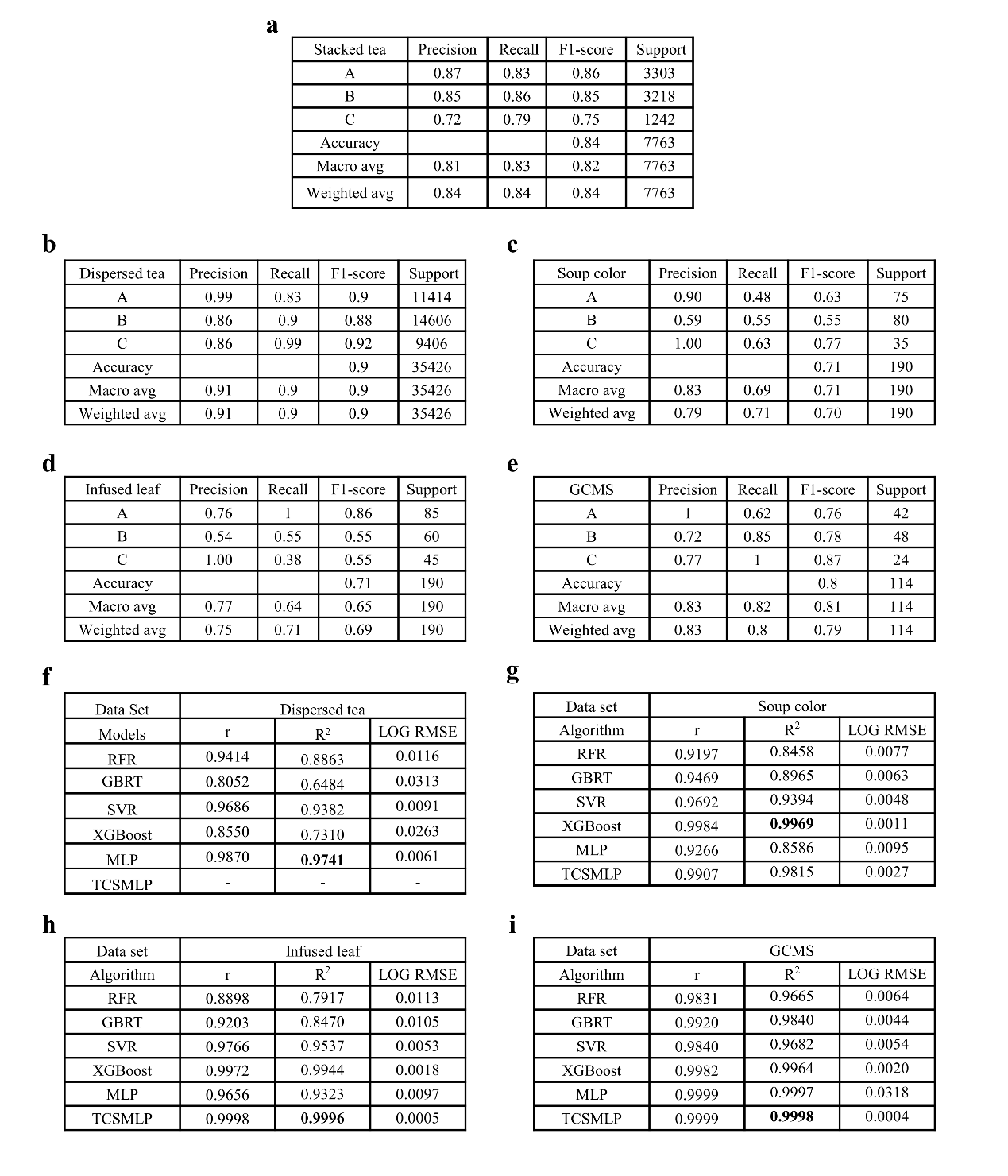


Supplementary Figure 3. Classification and regression performance metrics for different single-dimension datasets. (a–e) Classification metrics corresponding to the confusion matrices for stacked tea, dispersed tea, soup color, infused leaf, and the GC-MS-based aroma dataset, respectively. (f–i) Regression performance of six models (MLP, RFR, GBRT, SVR, XGBoost, and TCSMLP) on the dispersed tea, soup color, infused leaf, and GC-MS datasets, respectively, evaluated using r, R², and Log RMSE.

Fig. S4.


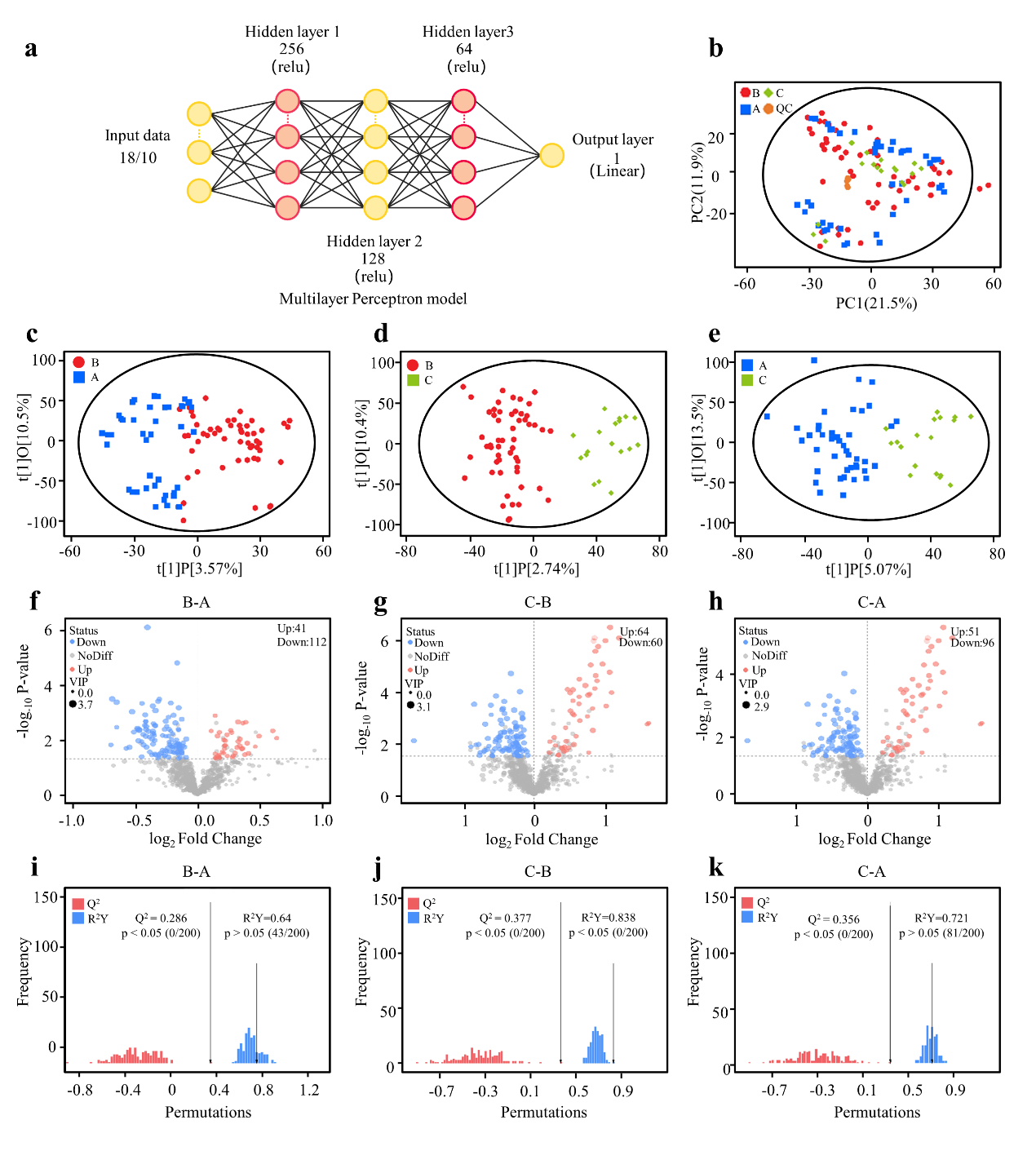


Supplementary Fig 4. Multivariate analysis of taste-related metabolomics data. (a) Architecture of the multilayer perceptron (MLP) model. (b) PCA score plot of the three tea Classs. (c–e) OPLS-DA score plots for pairwise comparisons between tea Classs: (c) Class B vs. Class A, (d) Class C vs. Class B, and (e) Class C vs. Class A. (f–h) Volcano plots showing differential metabolites for the same pairwise comparisons: (f) Class B vs. Class A, (g) Class C vs. Class B, and (h) Class C vs. Class A. (i–k) Permutation test results for the corresponding OPLS-DA models: (i) Class B vs. Class A, (j) Class C vs. Class B, and (k) Class C vs. Class A.

Fig. S5.


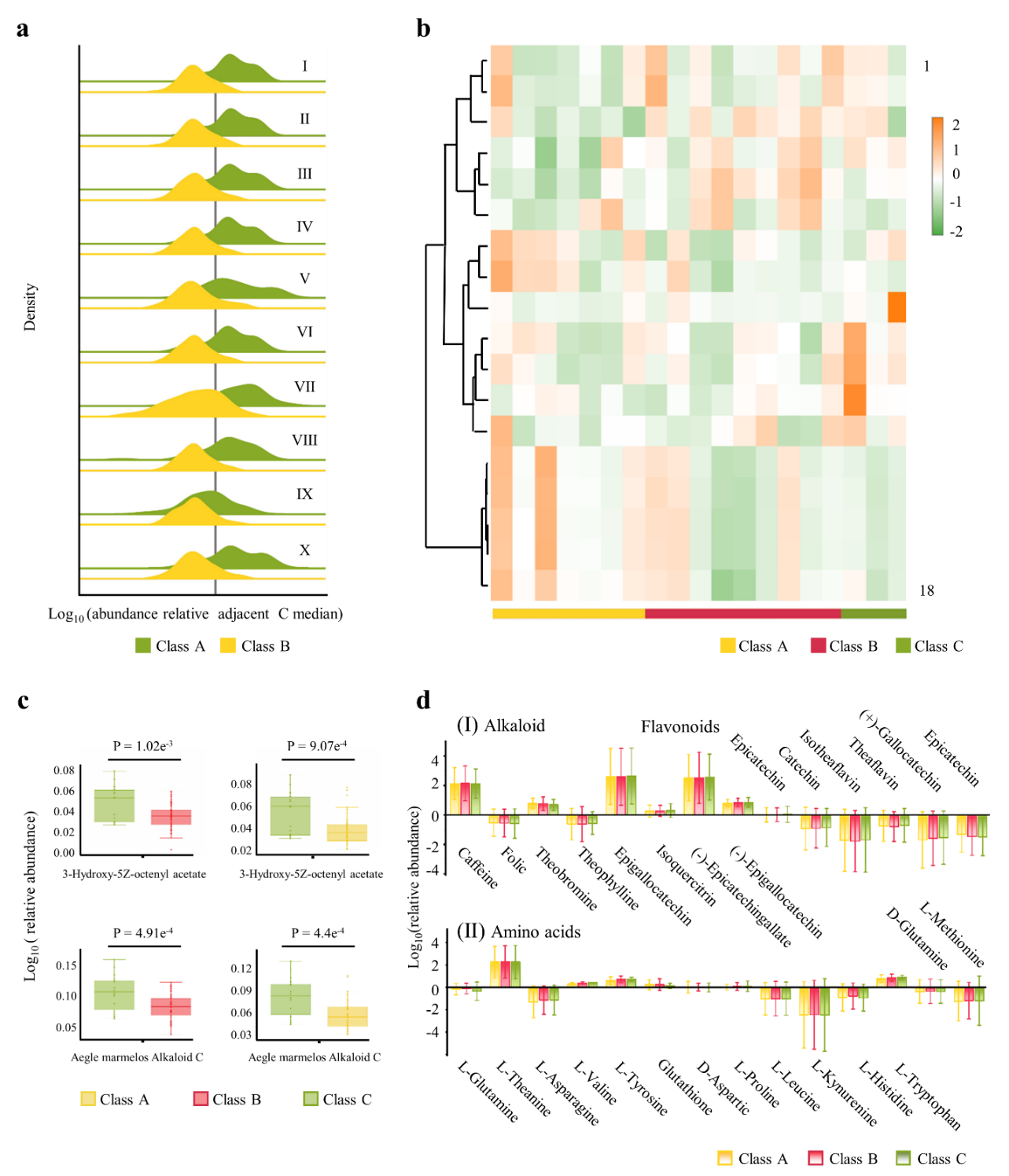


Supplementary Figure 5. Non-targeted metabolomics analysis of tea samples from different grades. (a) Density distributions of the relative abundances of 10 differential metabolites between matched Grade A and Grade B tea samples, expressed as ratios to the median relative abundance in Grade C samples. Metabolites I–X correspond to sucrose (I), trehalose (II), D-maltose (III), phlorin (IV), salvianolic acid A (V), 1,3,5-trihydroxybenzene (VI), 4-(2-aminophenyl)-2,4-dioxobutanoic acid (VII), 3-hydroxyadipic acid 3,6-lactone (VIII), hydroxykynurenine (IX), and 4-hydroxynornantenine (X). (b) Hierarchically clustered heatmap of 18 significantly differential metabolites among Grade A, Grade B, and Grade C tea samples, generated using Euclidean distance. Colors indicate relative metabolite abundance, with orange representing higher abundance and green representing lower abundance. (c) Relative abundances of 3-hydroxy-5Z-octenyl acetate and Aegle marmelos alkaloid C in tea samples from different grades. P values for specific independent pairwise comparisons (Class A vs. Class C, and Class B vs. Class C) were calculated using two-tailed Student’s t-tests, corresponding to the independent pairwise Volcano plot analyses. (d) Relative abundances of representative alkaloids (I), flavonoids, and amino acids (II). Tea class A, n = 42; Tea class B, n = 48; Tea class C, n = 24. Data are presented as mean ± SD.

Fig. S6.


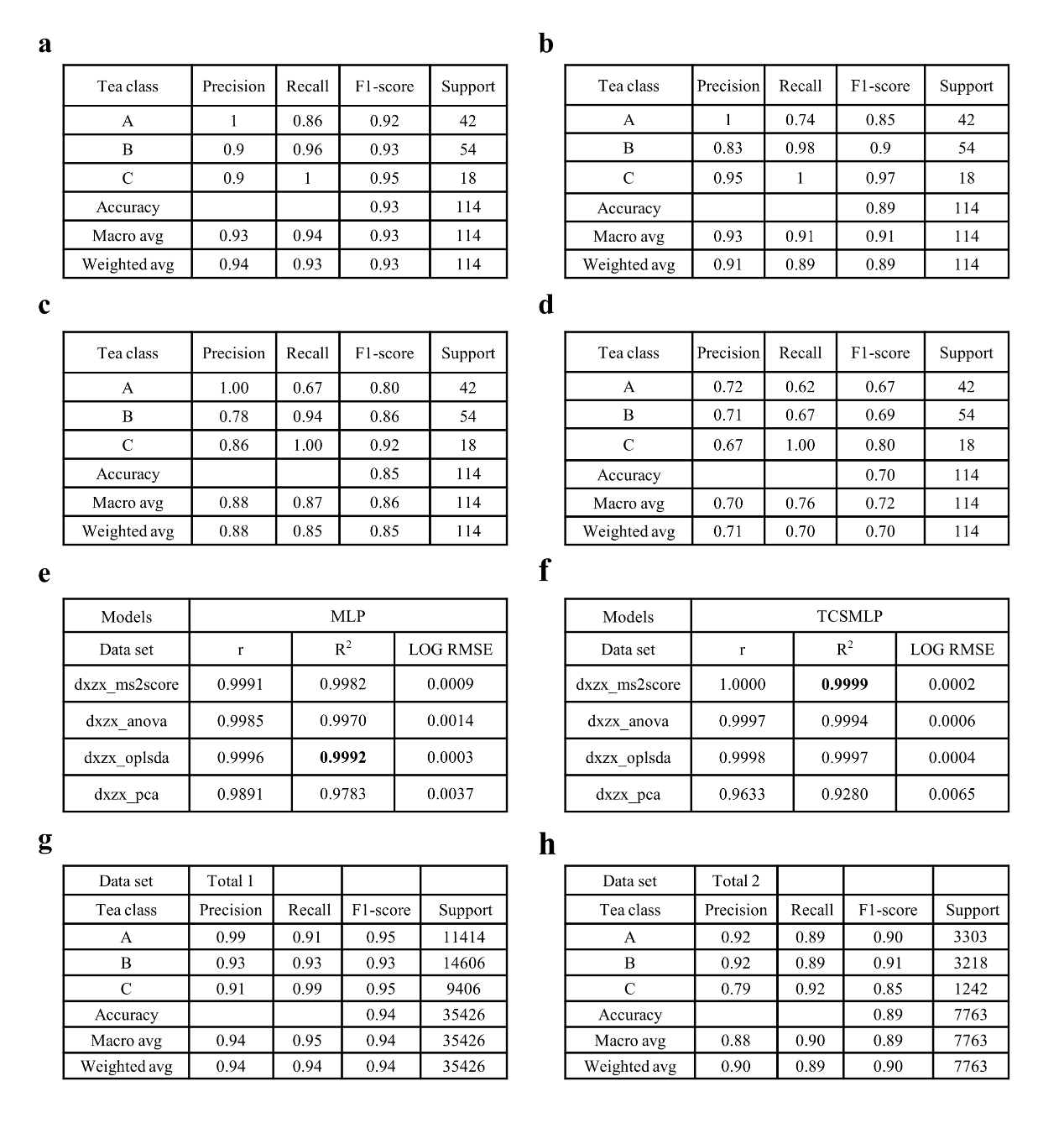


Supplementary Figure 6. Classification and regression performance metrics for different metabolomics datasets and weighted multimodal fusion results. (a–d) Classification metrics corresponding to the confusion matrices for the four metabolomics datasets: (a) DATA_MS2, (b) DATA_ANOVA, (c) DATA_OPLS-DA, and (d) DATA_PCA. (e, f) Regression performance of the MLP and TCSMLP models on different metabolomics datasets. (g, h) Classification metrics for the five-dimensional weighted fusion results obtained using two different integration strategies: (g) Classification result metrics for the 5-dimensional weighted confusion matrix: Appearance: feature extraction + MLP, soup color and infused leaf: XGBoost, GCMS metabolism: MLP, 5-dimensional weighted result. (h) Classification result metrics for the 5-dimensional weighted confusion matrix: Appearance: comment scoring + ResNet, soup color and infused leaf: XGBoost, GCMS metabolism: MLP, 5-dimensional weighted result. MLP. Support values are provided in each table.

Fig. S7.


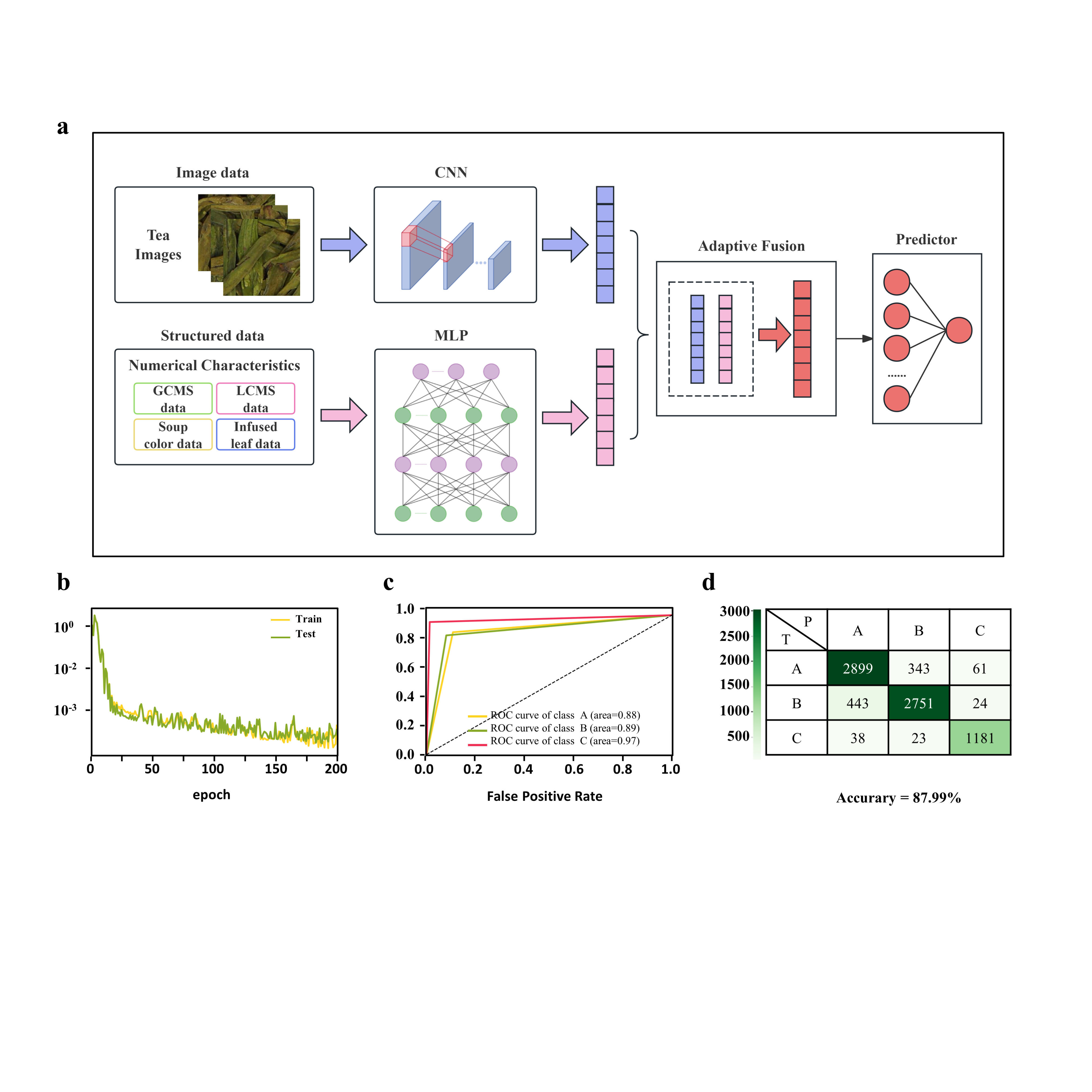


Supplementary Figure 7. Schematic diagram and performance evaluation of the feature-level fusion model. (a) Architecture of the feature-level fusion framework integrating image data and structured data. (b) Training and test loss curves over epochs. (c) ROC curves for the three classes. (d) Confusion matrix of the fused classification results. The ROC curves and confusion matrix were evaluated on the independent test set with n = 7,763 samples.

Tables S1. (Separate file) Tea sample information table and tea picture quantity table

Tables S2. (Separate file) Sensory evaluation results of tea (scores and comments)

Tables S3. (Separate file) Corresponding table of scoring and comments

Tables S4 (Separate file) Seven-dimensional training results of tea appearance trained with Resnet

Tables S5. (Separate file) Image feature extraction table of different grades of tea

Tables S6. (Separate file) Metabolome screening data results

Tables S7. (Separate file) The training results of the four datasets of metabolomics trained with five models respectively

Tables S8. (Separate file) The classification result metrics of the four-dimensional MLP optimized by CLIP

Data S1. (Separate file) Dispersed tea images features dataset

Data S2. (Separate file) Infused leaf features dataset

Data S3. (Separate file) Soup color features dataset

Data S4. (Separate file) GCMS features dataset

Data S5. (Separate file) All substances in metabolomics

Data S6. (Separate file) Substances identified by metabolomics
